# CD4^+^ and CD8^+^ T cells play a central role in a HDM driven model of allergic asthma

**DOI:** 10.1186/s12931-016-0359-y

**Published:** 2016-04-25

**Authors:** Kristof Raemdonck, Katie Baker, Nicole Dale, Eric Dubuis, Fisnik Shala, Maria G. Belvisi, Mark A. Birrell

**Affiliations:** Division of Airway Disease, Respiratory Pharmacology, National Heart & Lung Institute, Faculty of Medicine, Imperial College London, Exhibition Road, London, SW7 2AZ UK; MRC and Asthma UK Centre in Allergic Mechanisms of Asthma, Imperial College London, London, UK; Department of Anatomy, Faculty of Medicine, University of Porto, Alameda Prof. Hernâni Monteiro, 4200-319 Porto, Portugal; Center for Health Technology and Services Research (CINTESIS), Faculty of Medicine, University of Porto, Rua Dr. Plácido da Costa, 4200-450 Porto, Portugal

**Keywords:** Asthma, T cells, Animal model, Lung, Inflammation

## Abstract

**Background:**

The incidence of asthma is increasing at an alarming rate and while the current available therapies are effective in the majority of patients they fail to adequately control symptoms at the more severe end of the disease spectrum. In the search to understand disease pathogenesis and find effective therapies animal models are often employed. As exposure to house dust mite (HDM) has a causative link, it is thought of as the allergen of choice for modelling asthma.

The objective was to develop a HDM driven model of asthmatic sensitisation and characterise the role of key allergic effector cells/mediators.

**Methods:**

Mice were sensitised with low doses of HDM and then subsequently challenged. Cellular inflammation, IgE and airway responsiveness (AHR) was assessed in wild type mice or CD4^+^/CD8^+^ T cells, B cells or IgE knock out mice.

**Results:**

Only those mice sensitised with HDM responded to subsequent low dose topical challenge. Similar to the classical ovalbumin model, there was no requirement for systemic alum sensitisation. Characterisation of the role of effector cells demonstrated that the allergic cellular inflammation and AHR was dependent on CD4^+^ and CD8^+^ T cells but not B cells or IgE. Finally, we show that this model, unlike the classic OVA model, appears to be resistant to developing tolerance.

**Conclusions:**

This CD4^+^/CD8^+^ T cell dependent, HDM driven model of allergic asthma exhibits key features of asthma. Furthermore, we suggest that the ability to repeat challenge with HDM means this model is amenable to studies exploring the effect of therapeutic dosing in chronic, established disease.

**Electronic supplementary material:**

The online version of this article (doi:10.1186/s12931-016-0359-y) contains supplementary material, which is available to authorized users.

## Background

Asthma is a chronic inflammatory disease of the airways characterised not only by cellular infiltration into the airways, but also by an accompanying increase in the sensitivity and response to contractile agents (airway hyperresponsiveness-AHR) and to allergen exposure (early and late asthmatic responses) [1, 2]. The global prevalence of asthma is increasing [3, 4], and the symptoms are often inadequately controlled [5]. Pre-clinical models which closely mimic clinical features of asthma are useful to improve our understanding of the mechanisms driving the disease and thereby the ability to identify novel therapeutic targets.

Rodent models of experimental allergic asthma to date have contributed greatly to our current understanding of the pathogenesis of the disease. However, one of the most widely used aeroallergens in model development, ovalbumin (OVA), is rarely implicated in clinical asthma and is often used in more acute challenge protocols due to the development of inhalation tolerance and a diminishing or complete abrogation of the airway inflammatory response following multiple challenges [6–9]. Conventional rodent OVA-models are thought to require co-administration of an adjuvant to establish successful sensitisation. Therefore OVA may not be the most disease relevant allergen. In contrast, exposure to house dust mite (HDM), the major source of allergen in house dust, has been shown to play an important role in asthma and also in other allergic diseases such as dermatitis and rhinitis [10–13]. In the clinic it has been shown that sensitisation to HDM is a strong predictor for asthma and there is a correlation between the level of HDM exposure and sensitisation [10, 11]. Because of its clear relevance to human disease, HDM has in recent years become the allergen of choice in the development of animal models of asthma often relying on chronic topical exposure without developing tolerance [14–19]. However, it is possible that in these models part of the inflammatory response is due to innate mechanisms in response to repeat inflammatory insults rather than allergic mechanisms [20, 21]. We propose that to fully understand the disease phenotype it is important to characterise the different stages of the inflammatory response in vivo. Therefore the aim of this study was to initially demonstrate this acute innate response and briefly investigate the mechanisms involved (i.e. Dectin 1/2 and TLR 2/4 receptors have been linked to this response) and then to go on to develop a murine HDM-driven allergic model based around a separate sensitisation and challenge protocol. Furthermore we also investigated the role of key allergic effector cells in features of allergic asthma using this model. We additionally aimed to compare key features of this model to a standard OVA-model and investigate its potential for chronic allergen exposures without the associated tolerance. We suggest the data presented here will help to elucidate the mechanisms driving the inflammation observed after acute and chronic HDM exposure and present targets for possible future therapeutic intervention.

## Methods

### Animals

Male C57BL/6 mice (16–20 g) were obtained from Harlan UK Limited (Bicester, UK) and housed with food and water supplied ad libitum for at least 5 days before beginning treatments. Genetically modified mice (knockout, KOs) were back-crossed at least 8 times and bred alongside the wild type mice: Dectin-1 ^-/-^, Dectin-2 ^-/-^, TLR2 ^-/-^, TLR4^-/-^, B cell^-/-^ (J_H_T mice), CD4 T-cell ^-/-^, CD8 T-cell ^-/-^ and IgE ^-/-.^ Wild-type (WT) mice on a C57bl/6 background were bred *in-house* to be used as comparators for the KO animals. Protocols were approved by the Animal Welfare and Ethical Review Body (Approval No. PPL 70/7212), and strictly adhered to the Animals (Scientific Procedures) Act 1986 UK Home Office guidelines and performed according to the ARRIVE guidelines. Dectin-1 and Dectin-2 KO mice were donated by Professor Yoichiro Iwakura, Tokyo University; TLR2, TLR4, B cell, CD4^+^ T cell, CD8^+^ T cell and IgE KO mice were obtained from the Swiss Immunological Mouse Repository (SwimMR).

### House dust mite extract

Purified HDM extract from *Dermatophagoides pteronyssinus* (*Der p*; lot number 124632; GREER laboratories, USA) with a known content of *Der p1* (12.76 μg/mg dry weight) was used in these experiments. The doses of HDM used in this manuscript refer to the amount of *Der p1* delivered.

### Materials

All agents were purchased from Sigma-Aldrich (Poole, UK) unless otherwise stated.

### Effect of acute house dust mite

WT mice, TLR2, TLR4, Dectin-1 and Dectin-2 KO mice were anaesthetised (4 % Isoflurane in oxygen for 3 min, Abbott Laboratories, UK) and challenged with vehicle (saline) or HDM extract (25 μg, intratracheally – selected from a previous dose response study). A second group of WT mice was challenged with protease inactivated HDM (by heating to 65 °C for 30 min as previously described [20, 22]). WT mice were culled with an overdose of sodium pentobarbitone (200 mg/kg, i.p.; Merial, France) 2, 6, 24, 48, 72 or 96 h after challenge and KO mice at 72 h after challenge. Inflammatory cell recruitment in the lung was assessed. Briefly, the trachea was cannulated and bronchoalveolar lavage fluid (BALF) was obtained via lavage with 0.3 ml RPMI 1640 (Invitrogen, UK) 3times. The lavage fluid was pooled. BALF total white blood cell counts were performed using an automated cell counter (Sysmex F-820, Sysmex UK Ltd). Cytospins of BALF was prepared by centrifugation of 100 μl in a cytospin (Shandon, UK) at 700 RPM for 5 min. Slides were subsequently fixed and stained using a Hema-Tek 2000 (Ames Co., Elkhart, IN) with a modified Wright-Giemsa stain. Differential counts on 200 cells were performed following standard morphological criteria and the percentage of eosinophils, neutrophils and lymphocytes were determined. After lavaging the airway the lung was removed and the weight recorded. The lung tissue was then finely chopped and underwent an enzymatic digest to extract white blood cells. Subsequent analysis of white blood cell numbers was performed as described above.

### Development of an allergic HDM driven asthma model

#### Selection of sensitising dose and sensitisation route

To determine an optimum sensitisation dose and route a regimen adapted from a previously described OVA model [23] was used. Topically sensitised WT mice received intranasal (i.n.) saline or HDM (50 μl; 0.005–500 μg/kg) on day 0 and 14. Systemically sensitised WT mice received intraperitoneal (i.p.) saline, Alum (diluted 1:1 with saline; Pierce Biotechnology Inc, USA), HDM (0.005–500 μg/kg,) or Alum with HDM (0.005–500 μg/kg) on day 0 and 14. On day 21, following a sodium pentobarbitone (200 mg/kg, i.p.) overdose, heparinised blood samples were taken by cardiac puncture. Plasma total IgE and IgG_1_, HDM specific IgE and IgG_1_ levels were measured by ELISA.

#### Selection of challenging dose

Male C57bl/6 mice were sensitised on day 0 and 14 with saline in Alum or HDM in Alum (0.5 μg/kg, i.p.). Mice were challenged i.n. with saline or HDM (50 μl; 0.125–125 μg/kg) on days 24–26. Mice were lavaged 3 days after the last challenge. BALF total and differential white blood cell counts were performed as described above.

#### Determining the need for Alum – HDM and OVA models

##### HDM model

Male C57bl/6 mice were sensitised on day 0 and 14 with saline, Alum, HDM (0.5 μg/kg, i.p.) or Alum plus HDM (0.5 μg/kg) and challenged i.n. once daily on days 24–26 with saline or HDM (50 μl; 1.25 μg/kg). BALF was collected 3 days after the last challenge and inflammatory cell burden determined.

In a parallel group of mice sensitised with HDM only (no alum) 3 days after the final HDM challenge, airway responsiveness to 5-HT was assessed as changes in enhanced pause (Penh – conscious animals) or airway resistance (in anaesthetised animals).

##### Does topical challenge lead to sensitisation?

Male C57bl/6 mice were give saline or HDM (i.n. or i.p.) and subsequently challenged with saline or HDM as described above. 3 days after the final challenge lungs were lavaged and inflammatory cell burden determined.

##### OVA model

Male C57bl/6 mice were sensitised with saline, OVA (10 μg/100 μl i.p.), Alum plus saline or Alum plus OVA (10 μg/100 μl i.p.) on days 0 and 14 and then challenged i.n. with saline or OVA (50 μg) on days 24–26. Three days after the final OVA challenge airway responsiveness to 5-HT was assessed as changes in enhanced pause (Penh – conscious animals). BALF was collected for assessment of inflammatory cell burden, plasma samples were harvested and levels of total and OVA-specific IgE assessed by ELISA.

#### Chronicity of the HDM model

Male C57bl/6 mice were sensitised on day 0 and 14 with saline or HDM (0.5 μg/kg, i.p.) and challenged i.n. with saline or HDM (50 μl; 1.25 μg/kg) 3 times a week on consecutive days (starting on day 24) for 5 weeks. 3 days after the final HDM challenge airway responsiveness to 5-HT and the levels of cellular inflammation in BALF were assessed as described above. Lung tissue was collected for pathological assessment (inflammatory score, PAS staining, collagen deposition and ASM area assessment). Briefly, lung tissue was fixed with formalin and embedded in paraffin and multiple 4 uM thick sections cut.

##### Inflammation staining

Slides were stained with aematoxylin and eosin (H + E) and placed on the stage of an Olympus BX51 fluorescence microscope coupled to a QICAM 1394 camera and colour images were taken. Analysis was done by a trained person blinded to prior treatment. Six random areas were selected and each area was scored for inflammatory burden (0 to 5) and the data was averaged for each slide and then for each n number in the group.

##### Mucus production assessment

The slides were stained with Periodic acid–Schiff (PAS – mucus appears a bright purple-magenta colour). Assessment was performed using the protocol outlined above.

##### Smooth Muscle Area (SMA)

Alpha actin was stained using a primary antibody (Ab-5694, Abcam) and a secondary antibody (Ab 111-066-003, Jackson Labs). The staining was revealed using the peroxydase kit from vector laboratories (DAB Peroxidase (HRP) Substrate Kit) without Nickel (alpha actin stained in brown). The slides were counterstained with haematoxylin (blue). Analysis was performed as described above and expressed as SMA per airway perimeter.

##### Collagen deposition assessment

Slides were stained with Sirius Red and counter stained with haematoxylin. Slides were mounted on an inverted microscope (Zeiss AxioVert 200 M) equipped with a circularly polariser and coupled to a Hamamatsu EM-CCD C9100-02 camera. Images were taken in grey tone under polarised light were collagen stained with Sirius red appears brightly. Analysis was performed as above and expressed as an area of collagen per airway perimeter.

### Characterisation of the allergic HDM driven asthma model

#### Profiling a clinically relevant therapy

Mice were sensitised on day 0 and 14 with saline or HDM (0.5 μg/kg) i.p. and challenged i.n. with saline or HDM (50 μl; 1.25 μg/kg) on days 24–26. Mice received an oral dose of vehicle (0.5 % methylcellulose plus 0.2 % tween 80 in water, 10 ml/kg) or budesonide (3 mg/kg) twice per day on days 24–28 and also a final dose on day 29. 3 days after the final HDM challenge airway responsiveness to 5-HT and the levels of cellular inflammation in BALF were assessed as described above.

#### Determine the role of key allergic effector cells/mediator

WT, CD4^+^ T cell, CD8^+^ T cell, B cell or IgE KO mice were sensitised on day 0 and 14 with saline or HDM (0.5 μg/kg, i.p.) and challenged i.n. with saline or HDM (50 μl; 1.25 μg/kg) on days 24–26. 3 Days after the final HDM challenge airway responsiveness to 5-HT and the levels of cellular inflammation in BALF were assessed as described above.

### Data analysis and statistics

Data was expressed as mean ± S.E.M of n observations. A *p* value < 0.05 was taken as statistically significant, the test used is indicated in the figure legends.

## Results

### Acute HDM challenge

**Fig. 1:**
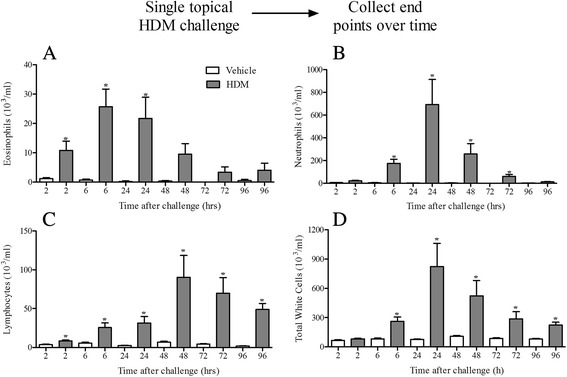
Effect of acute HDM dosing. Naïve male C57bl/6 mice were anaesthetised (4 % isoflurane in oxygen for 3 min) and challenged with i.t. saline or HDM. 2, 6, 24, 48, 72 and 96 h after challenge the lungs were lavaged and BALF eosinophil (**a**), neutrophil (**b**), lymphocyte (**c**) and total white cells (**d**) numbers were determined. Data (*n* = 6) expressed as mean cell numbers (10^3^/ml) ± S.E.M. *p < 0.05 vs. respective saline challenged time matched controls, Mann-Whitney *U*-test

Single topical challenge with HDM (25 μg) caused an inflammatory signal in naïve, healthy male mice, lasting up to 96 h after challenge. We observed increases in neutrophil numbers accompanied by significant increases in eosinophil, neutrophil and lymphocyte numbers (Fig. 1a–d) with a similar pattern observed in the lung tissue (data not shown). Various mechanisms have been implicated in HDM-driven inflammatory responses including the innate protease activity of the allergen [24, 25] and the activation of various pattern recognition receptors including TLR2, TLR4, dectin-1 and dectin-2 via the extract’s co-contaminants such as lipopolysaccharide and β-glucan moieties [26–30]. When we exposed mice deficient in TLR2, TLR4, Dectin-1 or Dectin-2 to a single HDM challenge, only TLR2 and Dectin-2 KO mice showed a reduced lung tissue inflammatory cell infiltration or a reduction in lung tissue weight (Additional file 1: Figure S1). No changes in BALF inflammatory cell numbers were observed. Due to this direct innate inflammatory response to the HDM extract we wanted to develop a murine model with an allergic phenotype i.e. where only allergen sensitised animals respond to allergen challenge.

### Development of an allergic HDM driven asthma model

#### Selection of optimum sensitising route and dose

**Fig. 2:**
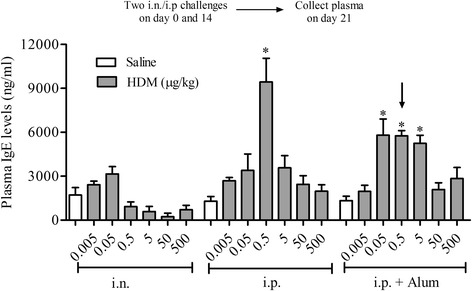
Selection of optimum sensitising route and dose. Male C57bl/6 mice were sensitised with vehicle or HDM (0.005–500 μg/kg HDM) either i.n. or i.p. (with or without Alum). Levels of total IgE in plasma were measured by ELISA. The dose and route selected for further model investigation is indicated by the black arrow. Data (*n* = 6–12) expressed as mean total IgE levels (ng/ml) ± S.E.M. *p < 0.05 vs. respective saline challenged controls, Kruskal-Wallis one-way ANOVA followed by Dunn’s Multiple Comparison post-test

To optimise the sensitisation phase of the model, using IgE as a marker of successful sensitisation, plasma total IgE was measured in mice treated with various doses of HDM via the intranasal and intraperitoneal routes; the latter with or without Alum. Systemic sensitisation is favoured in the classical OVA-models, while topical sensitisation with or without the addition of Alum is preferred in HDM-driven models. Our results show that topical i.n. sensitisation with HDM failed to induce any changes in total IgE compared to the respective controls (Fig. 2). In contrast, a bell-shaped increase in total IgE was observed with increasing HDM dose in systemically sensitised mice both with and without the addition of Alum compared to their respective controls (Fig. 2). This increase in total IgE was statistically significant in mice sensitised with 0.5 μg/kg HDM (both with and without Alum). At this sensitisation dose we also observed increases in HDM specific IgE, total IgG_1_ and HDM specific IgG_1_ in the plasma (Additional file 2: Figure S2). For this reason this dose was selected as the optimum dose to achieve allergic sensitisation in this model.

#### Selection of optimum challenging dose

**Fig. 3:**
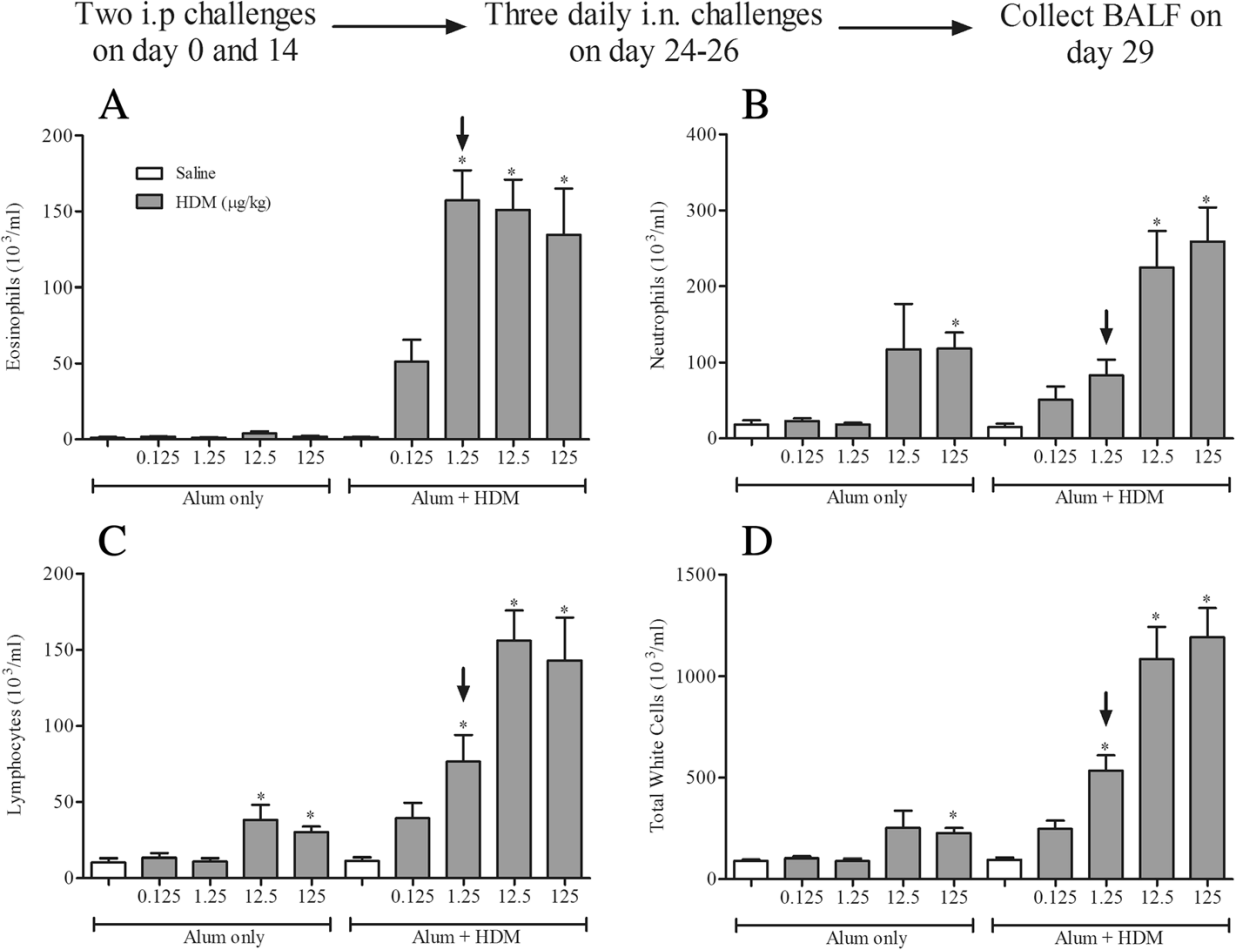
Selection of optimum challenging dose. Male C57bl/6 mice were sensitised with saline or HDM in the presence of Alum and challenged i.n. with saline or HDM (0.125–125 μg/kg HDM). 72 h after challenge the lungs were lavaged and BALF eosinophil (**a**), neutrophil (**b**), lymphocyte (**c**) and total white cell (**d**) numbers were determined. The dose selected for further model investigation is indicated by the black arrow. Data (*n* = 7–8) expressed as mean cell numbers (10^3^/ml) ± S.E.M. *p < 0.05 vs. relevant saline-challenged controls, Kruskal-Wallis one-way ANOVA followed by Dunn’s Multiple Comparison post-test

In this next phase we performed a dose response to intranasal allergen challenge in sensitised mice to establish a challenge dose which caused airway inflammation only in mice previously sensitised to HDM. As previous results suggested (Fig. 1), high doses of HDM (12.5 and/or 125 μg/kg HDM) significantly increased the number of BALF lymphocytes and neutrophils compared to saline-challenged controls without the need for prior allergen sensitisation (Fig. 3b and c). In contrast, low dose i.n. HDM challenge (1.25 μg/kg) in allergen sensitised mice caused a significant increase in BALF eosinophil, lymphocyte and neutrophil numbers, but importantly was not observed in mice which had not previously been exposed to HDM (Fig. 3). Due to this requirement of prior sensitisation to elicit an inflammatory response, the dose of 1.25 μg/kg HDM was selected as the challenge dose for the allergic HDM model. This dose induced sub-maximal levels of cellular inflammation in the BALF characterised by an influx of eosinophils, lymphocytes and macrophages without an accompanying non-allergic cellular inflammation.

#### Does topical challenge lead to sensitisation?

As we show in Fig. 2, in our hands intranasal sensitisation did not result in measurable changes in the levels of plasma IgE. The current trend in HDM model development has been to adopt experimental protocols employing repeated topical intranasal sensitisation with HDM followed by repeated topical intranasal HDM challenge, rather than the more traditional approach with distinct sensitisation and challenge phases. In our hands however, intranasal HDM challenge in mice topically sensitised with HDM did not result in an inflammatory cell influx into the BALF (Additional file 3: Figure S3). Mice subjected to our systemic i.p. sensitisation and topical i.n. challenge protocol, as shown previously (Fig. 3), mounted a robust inflammatory response as measured by increases in the number of eosinophils, neutrophils and lymphocytes in BALF (Additional file 3: Figure S3).

#### Determining the need for Alum

**Fig. 4:**
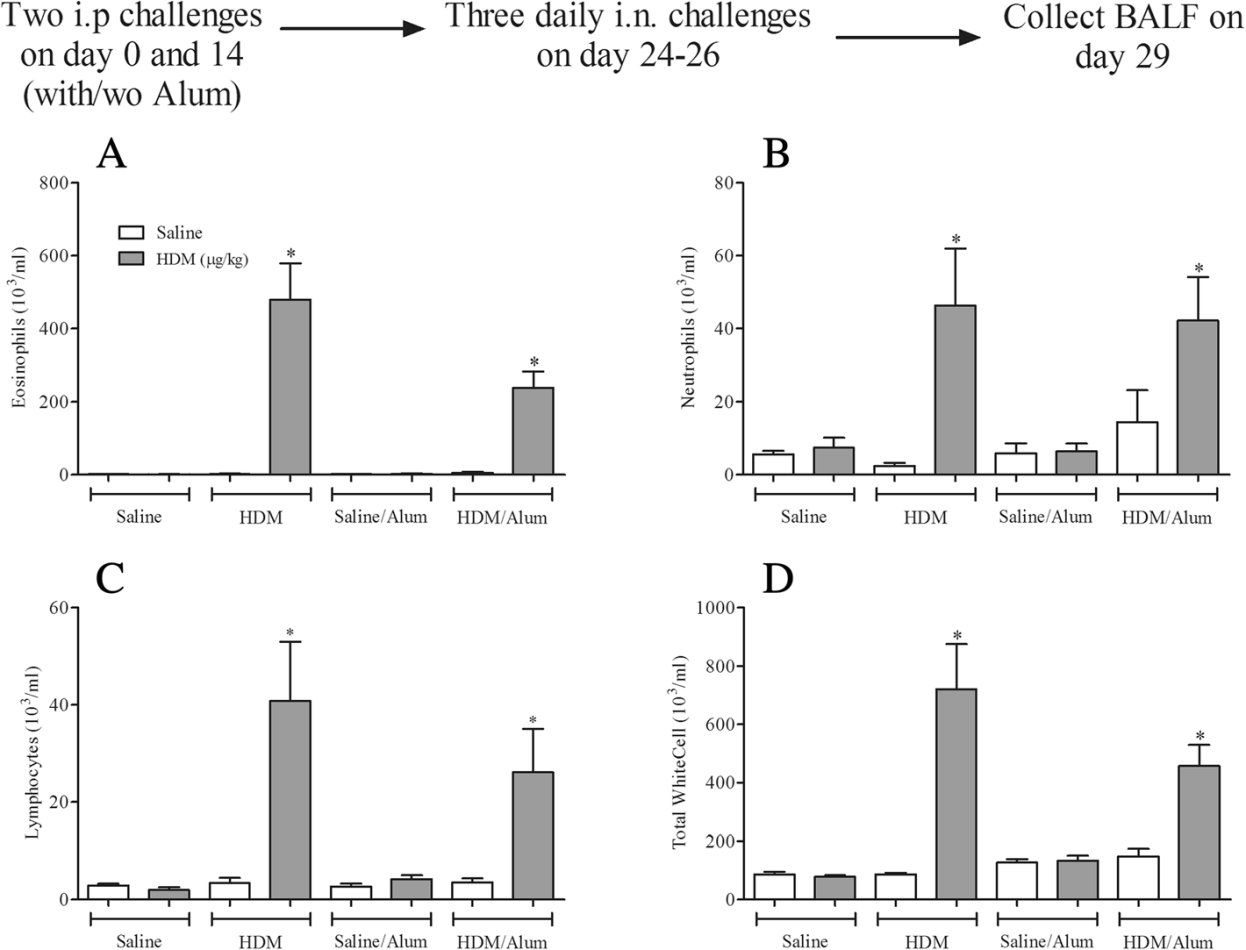
The requirement for Alum during sensitisation in the allergic inflammatory response to HDM challenge. Male C57bl/6 mice were sensitised with saline or HDM in the presence or absence of Alum. Mice were subsequently challenged with saline (open bars) or HDM (grey bars). 3 days after challenge the lungs were lavaged and BALF eosinophil (**a**), neutrophil (**b**), lymphocyte (**c**) and total white cell (**d**) numbers were determined. Data (*n* = 6) expressed as mean cell numbers (10^3^/ml) ± S.E.M. *p < 0.05 vs. relevant HDM sensitised/saline-challenged controls, Mann-Whitney *U*-test

As was shown in Fig. 2 an exogenous adjuvant was not necessary to achieve an increase in plasma total IgE after systemic sensitisation with HDM. The question remained as to whether Alum actually is needed during sensitisation in this model in order for an allergic response to occur after HDM challenge. Interestingly, i.n HDM challenge (1.25 μg/kg) in mice systemically sensitised to HDM with or without Alum induced statistically significant increases in the levels of BALF eosinophils, lymphocytes and neutrophils (Fig. 4). This clearly shows that Alum is not required to sensitise the mice to respond to a subsequent HDM challenge. Therefore, all experiments looking into the AHR, key effectors cells and mediators were performed without the use of the exogenous adjuvant Alum. In an OVA-dependent allergic mouse model of asthma, adopting the same sensitisation and challenge protocol used in the HDM-model, we show that despite the widespread use of adjuvants in conjunction with this allergen, Alum was not required to induce an allergic inflammatory response in the BALF characterised by an influx of eosinophils, neutrophils and lymphocytes (Additional file 4: Figure S4). In addition, OVA was able, without the addition of Alum, to induce robust total and OVA-specific IgE plasma levels (Additional file 5: Figure S5A and B) and AHR to 5-HT (Additional file 5: Figure S5C).

#### Airway function in HDM model

**Fig. 5:**
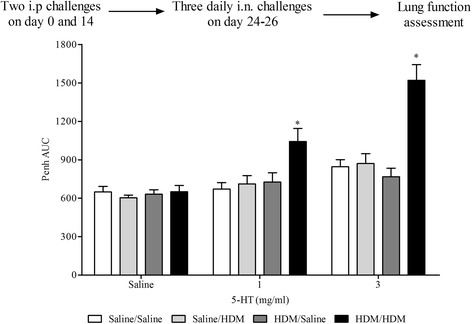
Effect of HDM sensitisation and challenge on airway responsiveness to inhaled 5-HT. Male C57bl/6 mice were sensitised with saline or HDM (no Alum) and subsequently challenged with saline or HDM. Conscious mice were placed in WBP chambers 3 days after final HDM challenge and airway responsiveness to inhaled 5-HT was assessed as Penh. Data (*n* = 6) expressed as mean Penh AUC ± S.E.M. *p < 0.05 vs. HDM-sensitised/saline-challenged controls, Mann-Whitney *U*-test

Functional endpoints such as AHR are very important to determine the usefulness of preclinical models of asthma. AHR in this HDM model was assessed 3 days after final HDM challenge as increases in airflow obstruction to aerosolised 5-HT measured by Penh. In HDM-sensitised and -challenged conscious mice the increase in airway responses to inhaled 5-HT was significantly enhanced compared to HDM-sensitised and saline-challenged control mice (Fig. 5). This phenomenon was mirrored when we recorded changes in resistance after aerosolised 5-HT in anaesthetised mice (Additional file 6: Figure S6).

#### Chronic HDM exposure

**Fig. 6:**
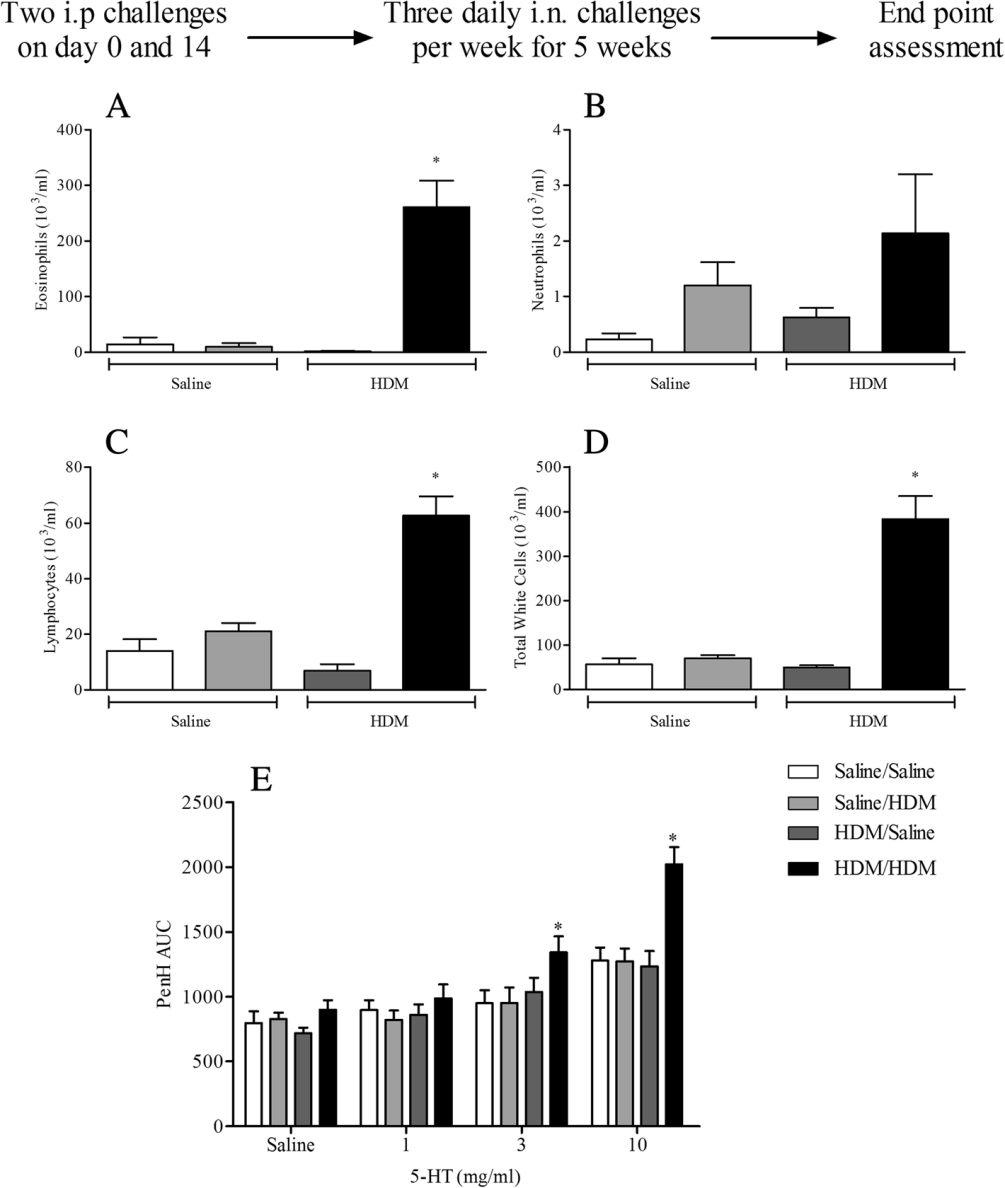
The effect of chronic HDM exposure on airway inflammation and airway responsiveness to inhaled 5-HT. Male WT C57bl/6 mice were sensitised with saline or HDM and subsequently challenged with saline or HDM 3 times a week for 5 weeks. Mice were placed in WBP chambers 3 days after final HDM challenge and airway responsiveness to inhaled 5-HT was assessed as Penh (**e**). Data (*n* = 6) expressed as mean Penh AUC ± S.E.M. Immediately after AHR assessment the lungs were lavaged and BALF eosinophil (**a**), neutrophil (**b**), lymphocyte (**c**) and total white cell (**d**) numbers were determined. Data (*n* = 6) expressed as mean cell number (10^3^/ml) ± S.E.M. *p < 0.05 vs. HDM sensitised, saline challenged controls, Mann-Whitney *U*-test

Chronic exposure of surrogate allergens such as OVA in animal models of asthma often leads to the development of tolerance [6–9]. This acquired tolerance hinders the investigation of underlying pathways and of the chronicity of the disease. Intermittent allergen exposure has been suggested to be able to overcome the development of tolerance [31, 32]. In our model, the exposure of previously sensitised mice to i.n. HDM 3 times a week for 5 weeks resulted in significantly increased eosinophil and lymphocyte numbers in BALF when compared to the respective control animals (Fig. 6a–c). While standard OVA models following chronic exposures often report diminished inflammatory responses, the increase in BALF inflammatory cells following chronic HDM challenge was persistent, possibly attributable to the intermittent challenge protocol employed. In addition, this inflammatory response was accompanied by a robust increase in airway responses to inhaled 5-HT (Fig. 6e). This inflammation in the BALF was associated with pathological changes in the lung tissue (Additional file 7: Figure S7).

### Characterisation of the allergic HDM driven asthma model

#### Profiling a clinically relevant therapy

**Fig. 7:**
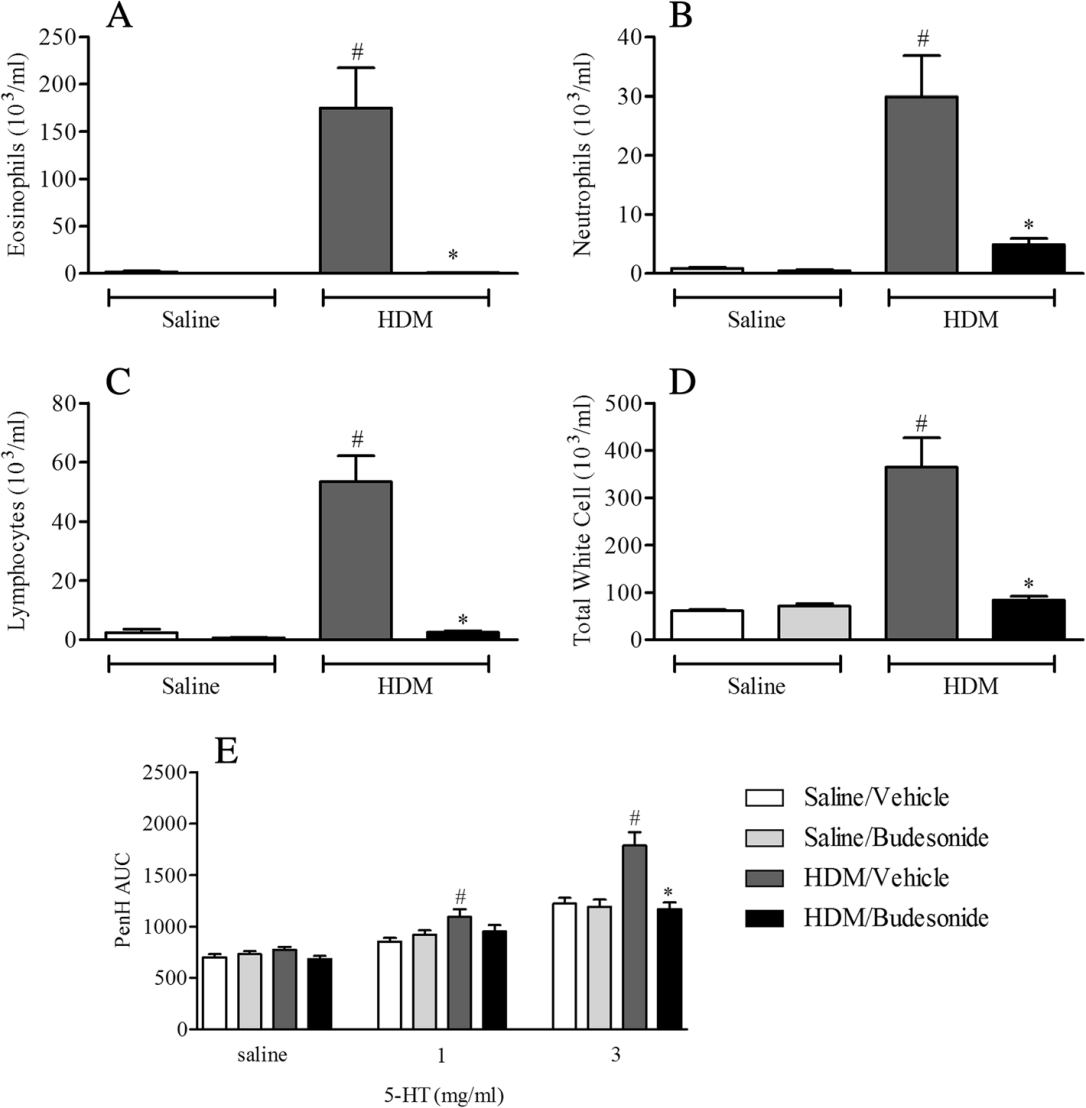
Effect of budesonide on HDM induced airway inflammation and airway responsiveness to inhaled 5-HT. Male C57bl/6 mice were sensitised with HDM, and subsequently challenged with saline or HDM. Mice were treated with vehicle (0.5 % methylcellulose plus 0.2 % tween80 in water) or budesonide (3 mg/kg). Mice were placed in WBP chambers 3 days after final HDM challenge and airway responsiveness to inhaled 5-HT was assessed as Penh (**e**). Data (*n* = 12) expressed as mean Penh AUC ± S.E.M. Immediately after AHR assessment the lungs were lavaged and BALF eosinophil (**a**), neutrophil (**b**), lymphocyte (**c**) and total white cell (**d**) numbers were determined. Data (*n* = 12) expressed as mean cell number (10^3^/ml) ± S.E.M. #*p* < 0.05 vs. relevant HDM-sensitised/saline-challenged controls, Mann-Whitney *U*-test. *p < 0.05 vs. relevant challenged/vehicle-treated controls, Mann-Whitney *U*-test

Exposing WT mice to the selected HDM sensitisation and challenge protocol led to significantly increased levels of neutrophils, eosinophils and lymphocytes in BALF and AHR in response to 5-HT (Figs. 4, 5 and 7). As expected pre-treatment with oral budesonide, a glucocorticosteroid, effectively and significantly inhibited the BALF inflammatory response and the increased airway response to 5-HT (Fig. 7a–e).

#### Determine the role of key allergic effector cells/mediators

**Fig. 8:**
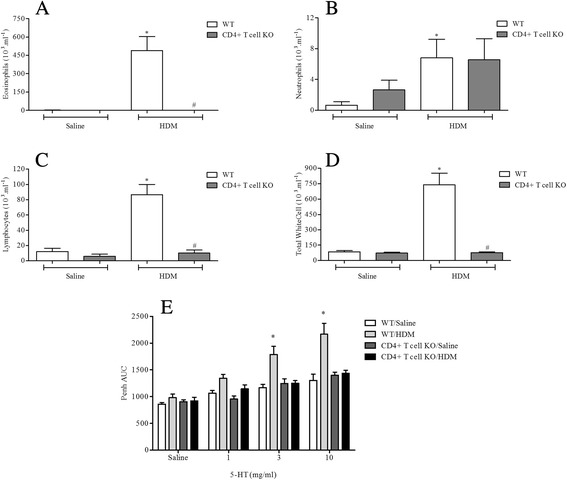
The role of CD4^+^ T-cells in HDM induced airway inflammation and airway responsiveness to inhaled 5-HT. Male WT C57bl/6 and CD4+ T-cell KO mice were sensitised with HDM and subsequently challenged with saline or HDM. Mice were placed in WBP chambers 3 days after final HDM challenge and airway responsiveness to inhaled 5-HT was assessed as Penh (**e**). Data (*n* = 7–8) expressed as mean Penh AUC ± S.E.M. Immediately after AHR assessment the lungs were lavaged and BALF eosinophil (**a**), neutrophil (**b**), lymphocyte (**c**) and total white cell (**d**) numbers were determined. Data (*n* = 7–8) expressed as mean cell number (10^3^/ml) ± S.E.M. *p < 0.05 vs. relevant saline-challenged controls, Mann-Whitney *U*-test. #*p* < 0.05 vs. challenged WT controls, Mann-Whitney *U*-test

**Fig. 9:**
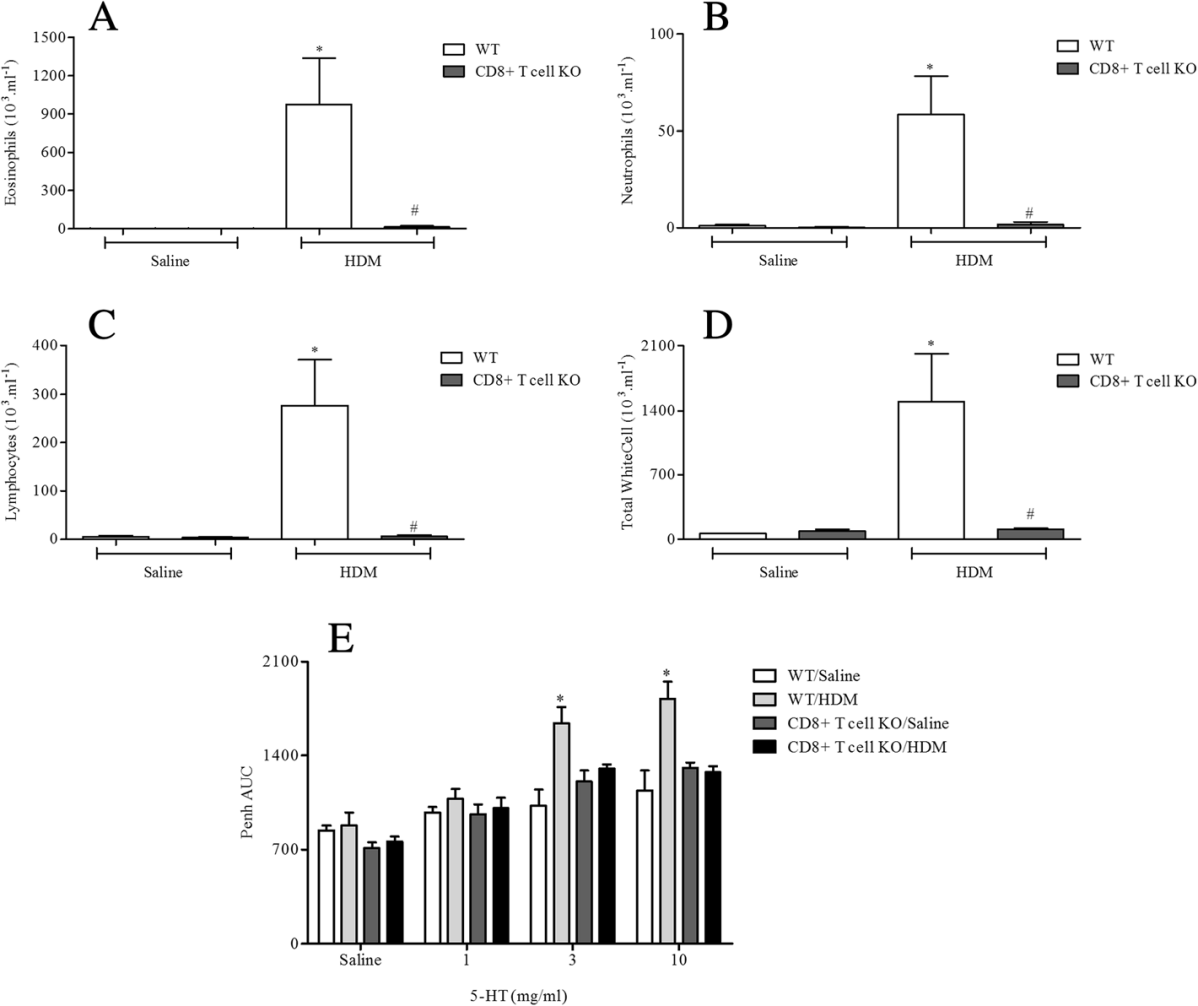
The role of CD8^+^ T-cells in HDM induced airway inflammation and airway responsiveness to inhaled 5-HT. Male WT C57bl/6 and CD8^+^ T-cell KO mice were sensitised with HDM and subsequently challenged with saline or HDM. Mice were placed in WBP chambers 3 days after final HDM challenge and airway responsiveness to inhaled 5-HT was assessed as Penh (**e**). Data (*n* = 6–7) expressed as mean Penh AUC ± S.E.M. Immediately after AHR assessment the lungs were lavaged and BALF eosinophil (**a**), neutrophil (**b**), lymphocyte (**c**) and total white cell (**d**) numbers were determined. Data (*n* = 6–7) expressed as mean cell number (10^3^/ml) ± S.E.M. *p < 0.05 vs. relevant saline-challenged controls, Mann-Whitney *U*-test. #*p* < 0.05 vs. challenged WT controls, Mann-Whitney *U*-test

**Fig. 10:**
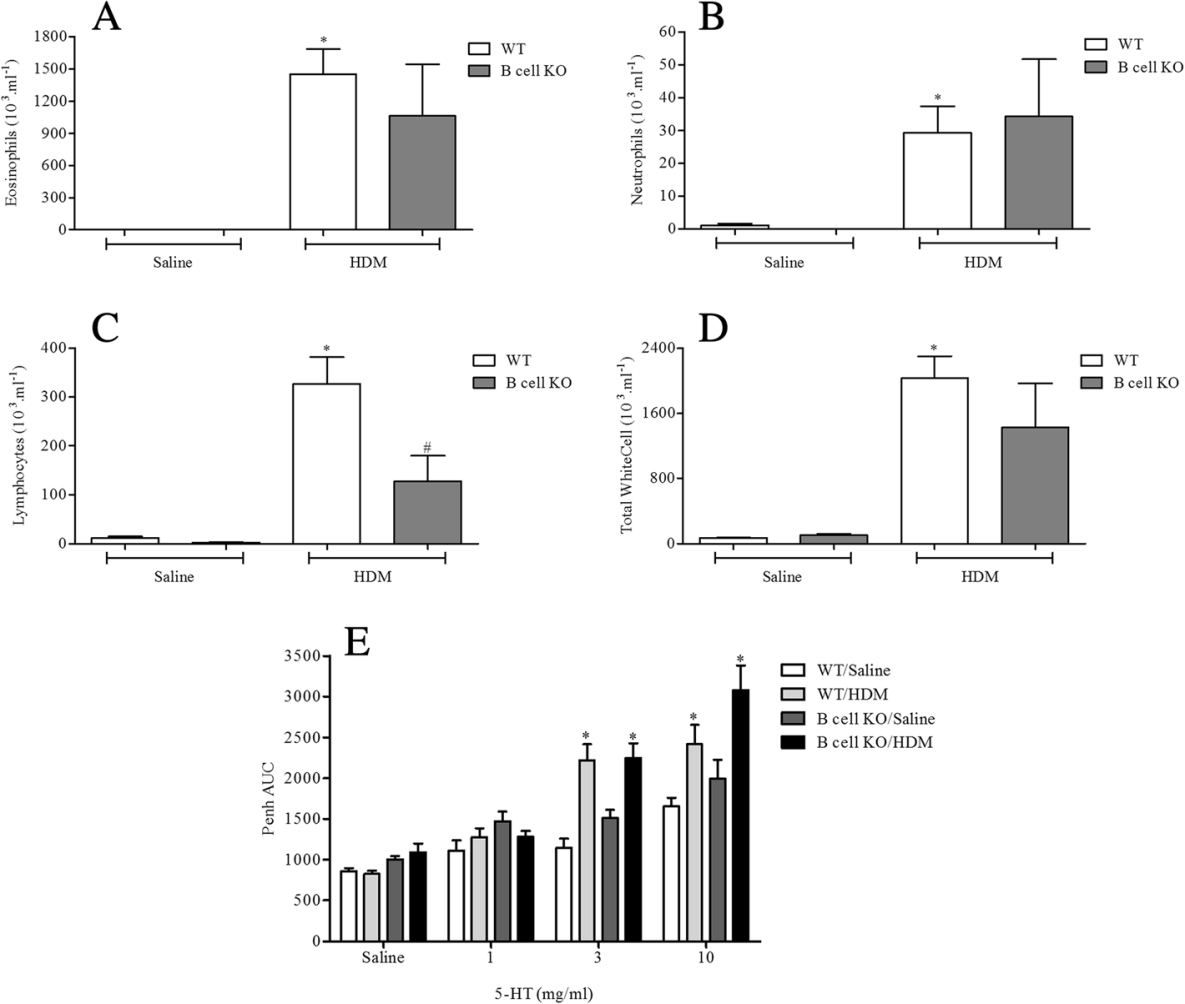
The role of B cells in HDM induced airway inflammation and airway responsiveness to inhaled 5-HT. Male WT C57bl/6 and B cell KO mice were sensitised with HDM and subsequently challenged with saline or HDM. Mice were placed in WBP chambers 3 days after final HDM challenge and airway responsiveness to inhaled 5-HT was assessed as Penh (**e**). Data (*n* = 6–9) expressed as mean Penh AUC ± S.E.M. Immediately after AHR assessment the lungs were lavaged and BALF eosinophil (**a**), neutrophil (**b**), lymphocyte (**c**) and total white cell (**d**) numbers were determined. Data (*n* = 6–9) expressed as mean cell number (10^3^/ml) ± S.E.M. *p < 0.05 vs. relevant saline-challenged controls, Mann-Whitney *U*-test. #*p* < 0.05 vs. challenged WT controls, Mann-Whitney *U*-test

**Fig. 11:**
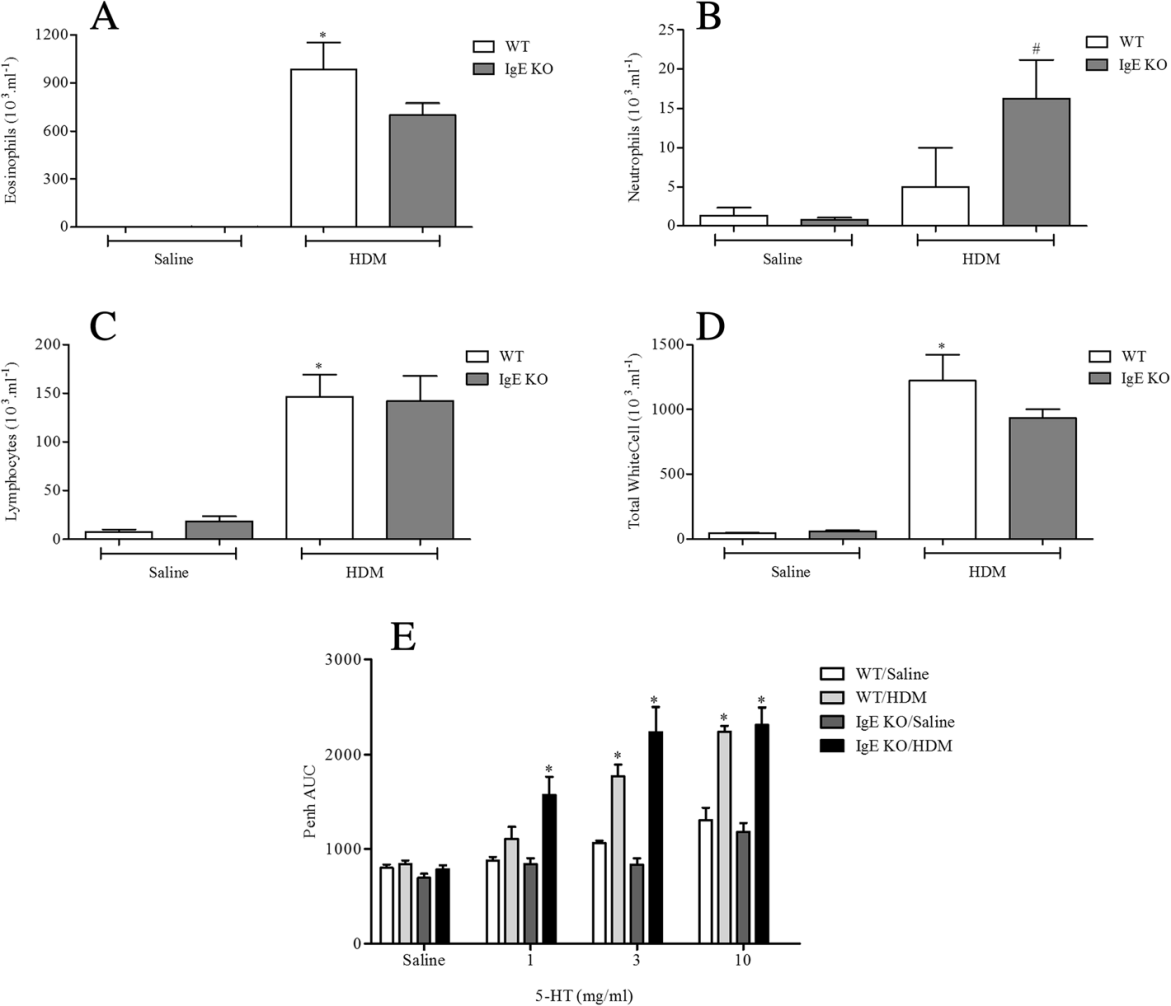
The role of IgE in HDM induced airway inflammation and airway responsiveness to inhaled 5-HT. Male WT C57bl/6 and IgE KO mice were sensitised with HDM and subsequently challenged with saline or HDM. Mice were placed in WBP chambers 3 days after final HDM challenge and airway responsiveness to inhaled 5-HT was assessed as Penh (**e**). Data (*n* = 8) expressed as mean Penh AUC ± S.E.M. Immediately after AHR assessment the lungs were lavaged and BALF eosinophil (**a**), neutrophil (**b**), lymphocyte (**c**) and total white cell (**d**) numbers were determined. Data (*n* = 8) expressed as mean cell number (10^3^/ml) ± S.E.M. *p < 0.05 vs. relevant saline-challenged controls, Mann-Whitney *U*-test. #*p* < 0.05 vs. challenged WT controls, Mann-Whitney *U*-test

Antigen challenge led to significantly increased levels of neutrophils, eosinophils and lymphocytes in BALF and AHR in response to 5-HT in WT mice (Figs. 8, 9, 10 and 11). In mice lacking functional CD4^+^ T cells or CD8^+^ T cells both the BALF cellular inflammation and the increased airway response to spasmogen were significantly reduced compared to allergic WT mice (Figs. 8 and 9a–d). In contrast, those mice missing functional B cells or with impaired IgE responses were able to mount an inflammatory response following HDM sensitisation and challenge similar to the response observed in allergic WT mice (Figs. 10 and 11a–d). This was associated with AHR to 5-HT (Figs. 10 and 11e). B cell KO mice did exhibit a reduced number of BALF lymphocytes (Fig. 10c), though this did not reach control levels and possibly reflects the lack of B cells themselves. These data suggest that while T cells are vital to the inflammation and AHR observed in this model, this response to HDM can occur independently from both B cells and IgE.

## Discussion

Pre-clinical models closely mimicking the cardinal features of asthma are useful in understanding the mechanisms driving the disease and can help in identifying novel therapeutic targets. Traditional models of allergic asthma involve the use of OVA along with adjuvants such as Alum and have been profiled extensively. Yet, these models have limitations. On the other hand, HDM is increasingly being used as a disease-relevant antigen in rodent models of allergic airway inflammation since it does not require an adjuvant and is not linked to the development of inhalation tolerance [15–18]. However, previous studies have suggested that part of the airway inflammation observed in response to topical HDM is due to innate rather than allergic mechanisms [20, 21]. Therefore, to fully understand the disease phenotype and extrapolate in vivo data to the clinic, we suggest it is necessary to clearly distinguish between the events underlying sensitisation and challenge, and characterise all stages of the allergic inflammatory response in vivo. In the present study, we describe an in vivo mouse model of HDM-induced allergic asthma with a distinct sensitisation and challenge phase characterised by allergen induced airway inflammation and AHR dependent on CD4^+^ and CD8^+^ T cells, but not B cells or IgE. This inflammation and AHR was sensitive to treatment with systemic steroids showing the model to be relevant to clinical asthma. In addition this model exhibits persistent airway inflammation and AHR following chronic exposure to the allergen.

A single topical exposure to HDM caused robust inflammatory cell recruitment into the mouse airway. Although the prominent cell type was neutrophils we could also detect an increase in eosinophils and lymphocytes (Fig. 1). This can be recapitulated in the rat [20] and thus far the mechanism underlying this innate response remains unclear. HDM extracts have been shown to contain various other contaminants capable of activating inflammatory pathways [33–35]. Our data exclude a role for HDM innate protease activity, TLR4 and Dectin-1 (Additional file 1: Figure S1) activation in this response despite previous studies implicating these factors in HDM-mediated inflammatory responses [24–26, 28], but show that there is a potential involvement of TLR2 and Dectin-2 (Additional file 1: Figure S1) in accordance with previous reports [27–30]. Although this falls outside the scope of the current publication, we suggest that further investigation of this underlying mechanism driving acute HDM-induced inflammation will lead to a better understanding of animal models, and could potentially uncover disease relevant mechanisms involved in the pathogenesis of asthma.

This apparent innate inflammatory response to HDM could confound the understanding of mechanisms underlying asthma-like features in animal models and complicate extrapolation of results to the clinic. We therefore developed a HDM-driven murine model in which airway inflammation and AHR only occurred in animals which had been previously sensitised to HDM. Using direct comparison studies we demonstrated that Alum during the sensitisation stage was not required for the allergic airway inflammation and AHR observed in this HDM model (Figs. 4 and 5). This is a great advantage as it avoids the use of an exogenous adjuvant and more closely resembles the allergenic cocktail to which patients are exposed. HDM has been demonstrated to possess innate adjuvant capacity and as such the fact that an exogenous adjuvant was not required for successful sensitisation or for the development of allergic asthma following allergen exposure, is not surprising. Interestingly, we also show that an adjuvant was not required for the production of OVA-specific IgE or the induction of airway inflammation and AHR in an OVA-dependent murine model (Additional file 4: Figure S4 and Additional file 5: Figure S5). This is surprising when one considers current dogma and that the majority of published models suggest that the OVA model requires the use of a systemic adjuvant to induce allergic sensitisation. Despite being widely criticised for not being clinically relevant and although adjuvant-free OVA models have been described [36–38] adjuvant dependent OVA-models continue to be used in the field. It has been noted that OVA is contaminated with lipopolysaccharide which might account for some of its auto-adjuvant properties [39].

Our results also highlight the importance of choosing the appropriate route for allergen delivery. Although several murine models utilising systemic sensitisation have been described [40, 41], the majority of research groups that utilise HDM-driven murine models currently favour topical sensitisation rather than systemic routes [14–19]. However, in contrast to these published models, this new HDM model uses systemic allergen sensitisation with a full HDM extract with all its associated contaminants without the need for an adjuvant. It is currently accepted that asthmatics become sensitised to HDM and other aeroallergens through the airways. However, several other mechanisms can lead to sensitisation and the development of allergic airways disease. Infants may have some features of allergy at birth through prenatal in-utero sensitisation [42–44]. In addition, in those atopic patients whereby atopic dermatitis is developed early on, followed by allergic rhinitis and subsequently atopic asthma later in life systemic sensitisation seems more likely, rather than sensitisation through airway exposure [45–47]. The fact that not every person exposed to HDM becomes sensitised and develops allergic asthma suggests that an animal model where allergen exposure only elicits a response in previously sensitised animals seems preferable. In contrast to reports that Balb/c mice can be primed to respond to HDM challenges via intranasal sensitisation [48] in our hands in this C57BL/6 murine model this did not occur (Fig. 2 and Additional file 3: Figure S3) and thus supports our use of systemic sensitisation during model development.

Due to the difficulty developing appropriate models capable of recapitulating key features of chronic asthma, the mechanisms underlying the chronicity of the disease have been far less investigated when compared to acute, immediate responses. The common allergen, OVA, frequently used in model development has been reported by many groups following chronic exposure protocols to result in inhalation tolerance and the abrogation of the inflammatory airway response [6–9]. It has been suggested that intermittent allergen exposures over prolonged periods of time do not result in the development of tolerance [31, 32]. Our data show that the administration of HDM over the course of 5 weeks in the absence of exogenous adjuvants does not lead to tolerance but a robust inflammatory response in HDM sensitised animals characterised by persistent airway eosinophilic, neutrophilic and lymphocytic airway infiltration accompanied by AHR to 5-HT (Fig. 6). It is plausible that the robust allergic response in our model is attributable to the non-continuous allergen exposure protocol. These results parallel data collected in those models employing chronic topical HDM challenges [18–23] and highlight the feasibility of using HDM to model chronic disease symptoms and the potential of the model to be used to assess the effect of new therapeutics dosed prophylactically and therapeutically in established ‘disease’ conditions.

HDM sensitisation induced the production of total and specific IgE (Fig. 2 and Additional file 2: Figure S2). Decades of research have implicated IgE-mediated responses in asthma and IgE is now a key target for asthma therapy. However, some have argued that increases in measurable IgE could be merely a result of asthma i.e. a marker of inflammation [49]. Additionally, studies utilising IgE directed therapy have shown mixed results [50]. Thus, IgE independent mechanisms have been suggested to also contribute to allergic disease [13, 51–53]. Through the use of KO mice deficient in IgE and functional B cells, our findings suggest that, at least in this model, IgE and B cells are not required for the generation of immune responses in animals systemically sensitised with and topically exposed to HDM (Figs. 10 and 11). This is in accordance with the suggestion that HDM can contribute to allergic disease independent of IgE, but is in contrast with a recent publication showing that mice, deficient in B cells, repeatedly exposed to an allergenic cocktail of HDM, *Alternaria* and *Aspergillus* exhibited reduced airway inflammation, lung pathology and AHR [54]. However, it is likely that the different allergens administered can act through different pathways some of which might indeed be independent of B cells and IgE production [55–58]. Furthermore, existing experimental model data, in contrast to these observations, suggested that the role of B cells in allergen induced immune responses may depend on the route of allergen delivery and on the type of allergen used [54, 58]. This could account for the differences with our model and the divergent data regarding the role of B cells in the regulation of immune responses to various allergens. Moreover, we show that the airway inflammatory response and associated AHR in this model is T cell dependent as CD8^+^ and CD4^+^ T cell KO mice failed to develop a response distinguishable from WT saline exposed control mice (Figs. 8 and 9). In the clinic elevated numbers of lymphocytes correlate with eosinophilia and asthma severity and this lymphocytosis has been shown to include both CD4^+^ and CD8^+^ T cells [59–61]. The CD4^+^ T cell is thought to be the dominant active T cell subtype in clinical allergic asthma as well as in animal models of asthma [57, 60, 62–64]. In contrast, the role of CD8^+^ T cells both in human asthma and animal models of allergic disease remains controversial, having been reported to be both protective or deleterious [65]. Supporting our data however, an increase in airway CD8^+^ T cells following allergen challenge predicts annual FEV_1_ decline in asthmatic patients [66, 67] and increased numbers can be found in patients following a fatal asthma attack [68, 69].

It is clear from published experimental data that CD4^+^ and CD8^+^ T cells can contribute to AHR development and allergic inflammation. However, the interpretation of their role and importance during sensitisation and/or challenge across various different models can vary largely. It is well established that CD4^+^ T cells are required during sensitisation [57, 70, 71]. Reconstitution of CD4^+^ T cells alone in RAG^-/-^ mice, deficient in both T-and B-cells, prior to sensitisation was sufficient for *Aspergillus fumigatus*-induced AHR and airway inflammation [57]. Interestingly, while CD8^+^ T cells were not required, this observation was also dependent on IL-4. Additionally, CD4^+^ T cell depletion before allergen challenge resulted in reduced AHR and airway eosinophilia [62] suggesting they are indeed crucial for the development of allergen induced airway disease. CD8^+^ T cells can differentiate, under the right conditions, in separate subsets [72–75]. It has been argued that these different subsets within different models could be responsible for the contradictory reports. Specific depletion of CD8^+^ T cells in sensitised rats enhanced the airway inflammation and related late asthmatic responses [76] and in addition augmented airway remodelling and mucus production. [77, 78]. Similarly, certain CD8^+^ T cell subsets have been shown to prevent OVA-induced inflammation in mice [79, 80]. Others have shown that CD4^+^, but not CD8^+^ T cells are required for allergic airway responses [57, 81]. In contrast, a body of work by Gelfand and colleagues [70, 71, 82, 83] using mainly a systemic sensitisation and topical challenge OVA mouse model of allergic asthma corroborates our results in this HDM model and highlights a role for both CD8^+^ and CD4^+^ T cells in AHR and airway inflammation. Using a variety of adoptive cell transfer studies and KO mice, they demonstrated the activation of allergen primed CD8^+^ T cells and IL-13 production within the airway [71, 83] critical for AHR and airway inflammation in this model. Additionally, their data suggest that CD4^+^ T cells and IL-4 are crucial during sensitisation for CD8^+^ T cell activation [70]. More recent publications also highlighted a possible role for IL-13 producing CD8^+^ T cell populations in augmented allergen driven inflammatory responses even in the absence of prior sensitisation [84]. It would be interesting to see whether similar pathways underlie the AHR and airway inflammation in our HDM model and merits further investigation. It is likely that a close interaction between CD4^+^ and CD8^+^ T cells is key to driving the allergic responses within our HDM model.

## Conclusion

In summary, we have developed a murine model of allergic asthma characterised by allergic airway inflammation and AHR which is dependent on prior allergen sensitisation without the need for an exogenous adjuvant. We show that this inflammation and AHR is dependent on CD4^+^ and CD8^+^ T cells and is persistent following multiple challenges. Our data reinforce the notion that T cells play a pivotal role in orchestrating pathological and physiological changes observed in human asthma. This newly developed model holds advantage over models that utilise topical administration in isolation in that sensitisation and challenge phases can be easily distinguished which allows separate interrogation. Additionally, with the possibility of repeat challenges this model could be useful in studies exploring therapeutic interventions in established chronic disease.

## Additional files

Additional file 1: Figure S1. Investigation of mechanism driving acute inflammatory response to HDM challenge. Naïve male C57bl/6, TLR2, TLR4, Dectin-1 and Dectin-2 KO mice were anaesthetised (4 % isoflurane in oxygen for 3 min) and challenged with i.t. vehicle (saline) or HDM (25 μg/mice in 50 μl saline). 48 h after challenge the lungs were lavaged, lung weight (A) determined, BALF (B) and lung tissue (C) total white blood cell numbers (D) were determined. Data (*n* = 7–8) expressed as means ± S.E.M. **p* < 0.05 vs saline challenged controls (Mann-Whitney test). #*p* < 0.05 vs. challenged WT controls, Mann-Whitney *U*-test. (DOC 185 kb) Additional file 2: Figure S2. Ig levels in response to sensitisation with HDM via different routes. Male C57bl/6 mice were sensitised with vehicle or HDM (5 μg/kg HDM) either i.n. or i.p. (with or without Alum). Levels of HDM-specific IgE (A), total IgG_1_ (B) and HDM-specific IgG_1_ (C) in plasma were measured by ELISA. Data (*n* = 6) expressed as means ± S.E.M. **p* < 0.05 vs. respective saline challenged controls, Mann-Whitney *U*-test. (DOC 183 kb) Additional file 3: Figure S3. The effect of intranasal HDM challenge in topically sensitised mice. Male C57bl/6 mice were sensitised i.n. or i.p. as indicated below the figures with saline or HDM. Mice were subsequently challenged with saline or HDM. 3 days after the last challenge lungs were lavaged and BALF eosinophil (A), neutrophil (B) and lymphocyte (C) numbers were determined. Data (*n* = 8–12) expressed as mean cell number (10^3^/ml) ± S.E.M. **p* < 0.05 vs. relevant saline sensitised controls, Mann-Whitney *U*-test. (DOC 109 kb) Additional file 4: Figure S4. The requirement for Alum during sensitisation in the allergic inflammatory response to OVA challenge. Male C57bl/6 mice were sensitised with saline or OVA made up in saline or Alum as indicated below the figure. Mice were subsequently challenged with saline (white bars) or OVA (grey bars). 3 days after the last challenge lungs were lavaged and BAL fluid eosinophil (A), neutrophil (B), lymphocyte (C) and total white blood cell (D) numbers were determined. Data expressed as mean cell number (10^3^/ml) ± S.E.M. (*n* = 7–9). **p* < 0.05 vs. relevant saline challenged controls, Mann-Whitney *U*-test. (DOC 177 kb) Additional file 5: Figure S5. Alum-independent OVA-induced airway hyperresponsiveness to 5-HT and IgE production. Male C57Bl/6 mice were sensitised with saline or OVA in the presence or absence of Alum and subsequently challenged with saline or OVA. Animals sensitised in the absence of Alum were placed in WBP chambers and airway responsiveness to 5-HT was assessed 3 days after final challenge (C). Data (*n* = 7–9) was expressed as mean Penh AUC ± S.E.M. Plasma levels of total IgE (A) and OVA-specific IgE (B) were assessed by ELISA 3 days after final OVA challenge. Data (*n* = 7–9) expressed as mean ± S.E.M. **p* < 0.05 vs. relevant OVA sensitised/saline challenged controls, Mann-Whitney *U*-test. (DOC 168 kb) Additional file 6: Figure S6. Effect of HDM sensitisation and challenge on airway responsiveness to inhaled 5-HT. Male C57bl/6 mice were sensitised with saline or HDM (no Alum) and subsequently challenged with saline or HDM. Anaesthetised mice were instrumented and airway responsiveness to inhaled 5-HT was assessed by changes in resistance. Data (*n* = 6) expressed as mean resistance ± S.E.M. (DOC 150 kb) Additional file 7: Figure S7. The effect of chronic HDM exposure on airway pathology. Male WT C57bl/6 mice were sensitised with saline or HDM and subsequently challenged with HDM 3 times a weeks for 5 weeks. Mice were culled 3 days after final HDM challenge and the lungs collected and assessed for inflammatory status: A) example H&E staining from the control group; B) example H&E staining from the allergic HDM challenged group; C) inflammatory cell burden score; D) mucus production (PAS staining) score; E) collagen deposition (Sirius Red staining) and F) airway smooth muscle area (alpha acting staining). Data (*n* = 6) expressed as mean ± S.E.M. #*p* < 0.05, Mann-Whitney *U*-test. (DOC 940 kb)

## Abbreviations

5-HT: 5-hydroxytryptamine
AHR: airway hyperresponsiveness
Alum: 20 mg/ml aluminium hydroxide and 20 mg/ml
AUC: area under the curve
BALF: bronchoalveolar lavage fluid
BSA: bovine serum albumin
CD: cluster of differentiation
ELISA: enzyme-linked immunosorbent assay
FEV_1_: forced expiratory volume in 1 s
HDM: house dust mite
HRP: horseradish peroxidase
i.n.: intranasal
i.p.: intraperitoneal
i.t.: intratracheal
IgE: immunoglobulin E
IL: interleukin
KO: knockout
OVA: ovalbumin
PBS: phosphate buffered saline
Penh: enhanced pause
S.E.M.: standard error of the mean
TLR: Toll-like receptor
TMB: 3,3′,5,5′-Tetramethylbenzidine
WBP: whole body plethysmograph
WT: wild type

## References

[1] Lemanske RF, Busse WW (2003). 6. Asthma. J Allergy Clin Immunol.

[2] Boulet LP, Gauvreau G, Boulay ME, O’Byrne P, Cockcroft DW (2007). The allergen bronchoprovocation model: an important tool for the investigation of new asthma anti-inflammatory therapies. Allergy.

[3] Masoli M, Fabian D, Holt S, Beasley R (2004). The global burden of asthma: executive summary of the GINA Dissemination Committee report. Allergy.

[4] Akinbami LJ, Moorman JE, Liu X (2011). Asthma prevalence, health care use, and mortality: United States, 2005–2009. Natl Health Stat Rep.

[5] Chapman KR, Boulet LP, Rea RM, Franssen E (2008). Suboptimal asthma control: prevalence, detection and consequences in general practice. Eur Respir J.

[6] Holt PG, Batty JE, Turner KJ (1981). Inhibition of specific IgE responses in mice by pre-exposure to inhaled antigen. Immunology.

[7] Wolvers DA, van der Cammen MJ, Kraal G (1997). Mucosal tolerance is associated with, but independent of, up-regulation Th2 responses. Immunology.

[8] Swirski FK, Gajewska BU, Alvarez D, Ritz SA, Cundall MJ, Cates EC, Coyle AJ, Gutierrez-Ramos JC, Inman MD, Jordana M, Stampfli MR (2002). Inhalation of a harmless antigen (ovalbumin) elicits immune activation but divergent immunoglobulin and cytokine activities in mice. Clin Exp Allergy.

[9] Van Hove CL, Maes T, Joos GF, Tournoy KG (2007). Prolonged inhaled allergen exposure can induce persistent tolerance. Am J Respir Cell Mol Biol.

[10] Maunsell K, Wraith DG, Cunnington AM (1968). Mites and house-dust allergy in bronchial asthma. Lancet.

[11] Platts-Mills TA, Wheatley LM (1996). The role of allergy and atopy in asthma. Curr Opin Pulm Med.

[12] Roche N, Chinet TC, Huchon GJ (1997). Allergic and nonallergic interactions between house dust mite allergens and airway mucosa. Eur Respir J.

[13] Hatzivlassiou M, Grainge C, Kehagia V, Lau L, Howarth PH (2010). The allergen specificity of the late asthmatic reaction. Allergy.

[14] Yu CK, Shieh CM, Lei HY (1999). Repeated intratracheal inoculation of house dust mite (Dermatophagoides farinae) induces pulmonary eosinophilic inflammation and IgE antibody production in mice. J Allergy Clin Immunol.

[15] Johnson JR, Wiley RE, Fattouh R, Swirski FK, Gajewska BU, Coyle AJ, Gutierrez-Ramos JC, Ellis R, Inman MD, Jordana M (2004). Continuous exposure to house dust mite elicits chronic airway inflammation and structural remodeling. Am J Respir Crit Care Med.

[16] Cates EC, Fattouh R, Johnson JR, Llop-Guevara A, Jordana M (2007). Modeling responses to respiratory house dust mite exposure. Contrib Microbiol.

[17] Southam DS, Ellis R, Wattie J, Inman MD (2007). Components of airway hyperresponsiveness and their associations with inflammation and remodeling in mice. J Allergy Clin Immunol.

[18] Gregory LG, Causton B, Murdoch JR, Mathie SA, O'Donnell V, Thomas CP, Priest FM, Quint DJ, Lloyd CM (2009). Inhaled house dust mite induces pulmonary T helper 2 cytokine production. Clin Exp Allergy.

[19] Chu DK, Al-Garawi A, Llop-Guevara A, Pillai RA, Radford K, Shen P, Walker TD, Goncharova S, Calhoun WJ, Nair P, Jordana M (2015). Therapeutic potential of anti-IL-6 therapies for granulocytic airway inflammation in asthma. Allergy Asthma Clin Immunol.

[20] De Alba J, Raemdonck K, Dekkak A, Collins M, Wong S, Nials AT, Knowles RG, Belvisi MG, Birrell MA (2010). House dust mite induces direct airway inflammation in vivo: implications for future disease therapy?. Eur Respir J.

[21] Birrell MA, Van Oosterhout AJ, Belvisi MG (2010). Do the current house dust mite-driven models really mimic allergic asthma?. Eur Respir J.

[22] Adam E, Hansen KK, Astudillo Fernandez O, Coulon L, Bex F, Duhant X, Jaumotte E, Hollenberg MD, Jacquet A (2006). The house dust mite allergen Der p 1, unlike Der p 3, stimulates the expression of interleukin-8 in human airway epithelial cells via a proteinase-activated receptor-2-independent mechanism. J Biol Chem.

[23] Birrell MA, De Alba J, Catley MC, Hardaker E, Wong S, Collins M, Clarke DL, Farrow SN, Willson TM, Collins JL, Belvisi MG (2008). Liver X receptor agonists increase airway reactivity in a model of asthma via increasing airway smooth muscle growth. J Immunol.

[24] King C, Brennan S, Thompson PJ, Stewart GA (1998). Dust mite proteolytic allergens induce cytokine release from cultured airway epithelium. J Immunol.

[25] Asokananthan N, Graham PT, Stewart DJ, Bakker AJ, Eidne KA, Thompson PJ, Stewart GA (2002). House dust mite allergens induce proinflammatory cytokines from respiratory epithelial cells: the cysteine protease allergen, Der p 1, activates protease-activated receptor (PAR)-2 and inactivates PAR-1. J Immunol.

[26] Hammad H, Chieppa M, Perros F, Willart MA, Germain RN, Lambrecht BN (2009). House dust mite allergen induces asthma via Toll-like receptor 4 triggering of airway structural cells. Nat Med.

[27] Barrett NA, Maekawa A, Rahman OM, Austen KF, Kanaoka Y (2009). Dectin-2 recognition of house dust mite triggers cysteinyl leukotriene generation by dendritic cells. J Immunol.

[28] Nathan AT, Peterson EA, Chakir J, Wills-Karp M (2009). Innate immune responses of airway epithelium to house dust mite are mediated through beta-glucan-dependent pathways. J Allergy Clin Immunol.

[29] Ryu JH, Yoo JY, Kim MJ, Hwang SG, Ahn KC, Ryu JC, Choi MK, Joo JH, Kim CH, Lee SN (2013). Distinct TLR-mediated pathways regulate house dust mite-induced allergic disease in the upper and lower airways. J Allergy Clin Immunol.

[30] Liu CF, Drocourt D, Puzo G, Wang JY, Riviere M (2013). Innate immune response of alveolar macrophage to house dust mite allergen is mediated through TLR2/-4 co-activation. PLoS One.

[31] Strickland DH, Stumbles PA, Zosky GR, Subrata LS, Thomas JA, Turner DJ, Sly PD, Holt PG (2006). Reversal of airway hyperresponsiveness by induction of airway mucosal CD4 + CD25+ regulatory T cells. J Exp Med.

[32] Schramm CM, Puddington L, Wu C, Guernsey L, Gharaee-Kermani M, Phan SH, Thrall RS (2004). Chronic inhaled ovalbumin exposure induces antigen-dependent but not antigen-specific inhalational tolerance in a murine model of allergic airway disease. Am J Pathol.

[33] Douwes J, Zuidhof A, Doekes G, van der Zee SC, Wouters I, Boezen MH, Brunekreef B (2000). (1-- > 3)-beta-D-glucan and endotoxin in house dust and peak flow variability in children. Am J Respir Crit Care Med.

[34] Thomas WR, Smith WA, Hales BJ, Mills KL, O'Brien RM (2002). Characterization and immunobiology of house dust mite allergens. Int Arch Allergy Immunol.

[35] Post S, Nawijn MC, Hackett TL, Baranowska M, Gras R, van Oosterhout AJ, Heijink IH (2012). The composition of house dust mite is critical for mucosal barrier dysfunction and allergic sensitisation. Thorax.

[36] Renz H, Smith HR, Henson JE, Ray BS, Irvin CG, Gelfand EW (1992). Aerosolized antigen exposure without adjuvant causes increased IgE production and increased airway responsiveness in the mouse. J Allergy Clin Immunol.

[37] Hessel EM, Van Oosterhout AJ, Hofstra CL, De Bie JJ, Garssen J, Van Loveren H, Verheyen AK, Savelkoul HF, Nijkamp FP (1995). Bronchoconstriction and airway hyperresponsiveness after ovalbumin inhalation in sensitized mice. Eur J Pharmacol.

[38] Besnard AG, Guillou N, Tschopp J, Erard F, Couillin I, Iwakura Y, Quesniaux V, Ryffel B, Togbe D (2011). NLRP3 inflammasome is required in murine asthma in the absence of aluminum adjuvant. Allergy.

[39] Eisenbarth SC, Piggott DA, Huleatt JW, Visintin I, Herrick CA, Bottomly K (2002). Lipopolysaccharide-enhanced, toll-like receptor 4-dependent T helper cell type 2 responses to inhaled antigen. J Exp Med.

[40] Tournoy KG, Kips JC, Schou C, Pauwels RA (2000). Airway eosinophilia is not a requirement for allergen-induced airway hyperresponsiveness. Clin Exp Allergy.

[41] Kelada SN, Wilson MS, Tavarez U, Kubalanza K, Borate B, Whitehead GS, Maruoka S, Roy MG, Olive M, Carpenter DE (2011). Strain-dependent genomic factors affect allergen-induced airway hyperresponsiveness in mice. Am J Respir Cell Mol Biol.

[42] Miller RL, Chew GL, Bell CA, Biedermann SA, Aggarwal M, Kinney PL, Tsai WY, Whyatt RM, Perera FP, Ford JG (2001). Prenatal exposure, maternal sensitization, and sensitization in utero to indoor allergens in an inner-city cohort. Am J Respir Crit Care Med.

[43] Hagendorens MM, Ebo DG, Bridts CH, Van de Water L, De Clerck LS, Stevens WJ (2004). Prenatal exposure to house dust mite allergen (Der p 1), cord blood T cell phenotype and cytokine production and atopic dermatitis during the first year of life. Pediatr Allergy Immunol.

[44] Peters JL, Suglia SF, Platts-Mills TA, Hosen J, Gold DR, Wright RJ (2009). Relationships among prenatal aeroallergen exposure and maternal and cord blood IgE: project ACCESS. J Allergy Clin Immunol.

[45] van der Hulst AE, Klip H, Brand PL (2007). Risk of developing asthma in young children with atopic eczema: a systematic review. J Allergy Clin Immunol.

[46] Kapoor R, Menon C, Hoffstad O, Bilker W, Leclerc P, Margolis DJ (2008). The prevalence of atopic triad in children with physician-confirmed atopic dermatitis. J Am Acad Dermatol.

[47] Spergel JM (2010). From atopic dermatitis to asthma: the atopic march. Ann Allergy Asthma Immunol.

[48] Phipps S, Lam CE, Kaiko GE, Foo SY, Collison A, Mattes J, Barry J, Davidson S, Oreo K, Smith L (2009). Toll/IL-1 signaling is critical for house dust mite-specific helper T cell type 2 and type 17 [corrected] responses. Am J Respir Crit Care Med.

[49] Sunyer J, Anto JM, Castellsague J, Soriano JB, Roca J (1996). Total serum IgE is associated with asthma independently of specific IgE levels. The Spanish Group of the European Study of Asthma. Eur Respir J.

[50] Avila PC (2007). Does anti-IgE therapy help in asthma? Efficacy and controversies. Annu Rev Med.

[51] Machado DC, Horton D, Harrop R, Peachell PT, Helm BA (1996). Potential allergens stimulate the release of mediators of the allergic response from cells of mast cell lineage in the absence of sensitization with antigen-specific IgE. Eur J Immunol.

[52] Haselden BM, Kay AB, Larche M (1999). Immunoglobulin E-independent major histocompatibility complex-restricted T cell peptide epitope-induced late asthmatic reactions. J Exp Med.

[53] Ali FR, Kay AB, Larche M (2007). Airway hyperresponsiveness and bronchial mucosal inflammation in T cell peptide-induced asthmatic reactions in atopic subjects. Thorax.

[54] Drake LY, Iijima K, Hara K, Kobayashi T, Kephart GM, Kita H (2015). B cells play key roles in th2-type airway immune responses in mice exposed to natural airborne allergens. PLoS One.

[55] Mehlhop PD, van de Rijn M, Goldberg AB, Brewer JP, Kurup VP, Martin TR, Oettgen HC (1997). Allergen-induced bronchial hyperreactivity and eosinophilic inflammation occur in the absence of IgE in a mouse model of asthma. Proc Natl Acad Sci U S A.

[56] Hamelmann E, Vella AT, Oshiba A, Kappler JW, Marrack P, Gelfand EW (1997). Allergic airway sensitization induces T cell activation but not airway hyperresponsiveness in B cell-deficient mice. Proc Natl Acad Sci U S A.

[57] Corry DB, Grunig G, Hadeiba H, Kurup VP, Warnock ML, Sheppard D, Rennick DM, Locksley RM (1998). Requirements for allergen-induced airway hyperreactivity in T and B cell-deficient mice. Mol Med.

[58] Hamelmann E, Tadeda K, Oshiba A, Gelfand EW (1999). Role of IgE in the development of allergic airway inflammation and airway hyperresponsiveness--a murine model. Allergy.

[59] Walker C, Kaegi MK, Braun P, Blaser K (1991). Activated T cells and eosinophilia in bronchoalveolar lavages from subjects with asthma correlated with disease severity. J Allergy Clin Immunol.

[60] Robinson DS, Bentley AM, Hartnell A, Kay AB, Durham SR (1993). Activated memory T helper cells in bronchoalveolar lavage fluid from patients with atopic asthma: relation to asthma symptoms, lung function, and bronchial responsiveness. Thorax.

[61] Ying S, Humbert M, Barkans J, Corrigan CJ, Pfister R, Menz G, Larche M, Robinson DS, Durham SR, Kay AB (1997). Expression of IL-4 and IL-5 mRNA and protein product by CD4+ and CD8+ T cells, eosinophils, and mast cells in bronchial biopsies obtained from atopic and nonatopic (intrinsic) asthmatics. J Immunol.

[62] Gavett SH, Chen X, Finkelman F, Wills-Karp M (1994). Depletion of murine CD4+ T lymphocytes prevents antigen-induced airway hyperreactivity and pulmonary eosinophilia. Am J Respir Cell Mol Biol.

[63] Robinson D, Hamid Q, Bentley A, Ying S, Kay AB, Durham SR (1993). Activation of CD4+ T cells, increased TH2-type cytokine mRNA expression, and eosinophil recruitment in bronchoalveolar lavage after allergen inhalation challenge in patients with atopic asthma. J Allergy Clin Immunol.

[64] Kon OM, Sihra BS, Compton CH, Leonard TB, Kay AB, Barnes NC (1998). Randomised, dose-ranging, placebo-controlled study of chimeric antibody to CD4 (keliximab) in chronic severe asthma. Lancet.

[65] Betts RJ, Kemeny DM (2009). CD8+ T cells in asthma: friend or foe?. Pharmacol Ther.

[66] van Rensen EL, Sont JK, Evertse CE, Willems LN, Mauad T, Hiemstra PS, Sterk PJ (2005). Bronchial CD8 cell infiltrate and lung function decline in asthma. Am J Respir Crit Care Med.

[67] Dakhama A, Collins ML, Ohnishi H, Goleva E, Leung DY, Alam R, Sutherland ER, Martin RJ, Gelfand EW (2013). IL-13-producing BLT1-positive CD8 cells are increased in asthma and are associated with airway obstruction. Allergy.

[68] O'Sullivan S, Cormican L, Faul JL, Ichinohe S, Johnston SL, Burke CM, Poulter LW (2001). Activated, cytotoxic CD8(+) T lymphocytes contribute to the pathology of asthma death. Am J Respir Crit Care Med.

[69] O'Sullivan SM (2005). Asthma death, CD8+ T cells, and viruses. Proc Am Thorac Soc.

[70] Koya T, Miyahara N, Takeda K, Matsubara S, Matsuda H, Swasey C, Balhorn A, Dakhama A, Gelfand EW (2007). CD8+ T cell-mediated airway hyperresponsiveness and inflammation is dependent on CD4 + IL-4+ T cells. J Immunol.

[71] Koya T, Matsuda H, Matsubara S, Miyahara N, Dakhama A, Takeda K, Gelfand EW (2009). Differential effects of dendritic cell transfer on airway hyperresponsiveness and inflammation. Am J Respir Cell Mol Biol.

[72] Croft M, Carter L, Swain SL, Dutton RW (1994). Generation of polarized antigen-specific CD8 effector populations: reciprocal action of interleukin (IL)-4 and IL-12 in promoting type 2 versus type 1 cytokine profiles. J Exp Med.

[73] Sad S, Marcotte R, Mosmann TR (1995). Cytokine-induced differentiation of precursor mouse CD8+ T cells into cytotoxic CD8+ T cells secreting Th1 or Th2 cytokines. Immunity.

[74] Cerwenka A, Carter LL, Reome JB, Swain SL, Dutton RW (1998). In vivo persistence of CD8 polarized T cell subsets producing type 1 or type 2 cytokines. J Immunol.

[75] Mittrucker HW, Visekruna A, Huber M (2014). Heterogeneity in the differentiation and function of CD8(+) T cells. Arch Immunol Ther Exp (Warsz).

[76] Isogai S, Jedrzkiewicz S, Taha R, Hamid Q, Martin JG (2005). Resident CD8+ T cells suppress CD4+ T cell-dependent late allergic airway responses. J Allergy Clin Immunol.

[77] Laberge S, Wu L, Olivenstein R, Xu LJ, Renzi PM, Martin JG (1996). Depletion of CD8+ T cells enhances pulmonary inflammation but not airway responsiveness after antigen challenge in rats. J Allergy Clin Immunol.

[78] Tsuchiya K, Isogai S, Tamaoka M, Inase N, Akashi T, Martin JG, Yoshizawa Y (2009). Depletion of CD8+ T cells enhances airway remodelling in a rodent model of asthma. Immunology.

[79] Ishimitsu R, Nishimura H, Yajima T, Watase T, Kawauchi H, Yoshikai Y (2001). Overexpression of IL-15 in vivo enhances Tc1 response, which inhibits allergic inflammation in a murine model of asthma. J Immunol.

[80] Sawicka E, Noble A, Walker C, Kemeny DM (2004). Tc2 cells respond to soluble antigen in the respiratory tract and induce lung eosinophilia and bronchial hyperresponsiveness. Eur J Immunol.

[81] Watanabe A, Mishima H, Renzi PM, Xu LJ, Hamid Q, Martin JG (1995). Transfer of allergic airway responses with antigen-primed CD4+ but not CD8+ T cells in brown Norway rats. J Clin Invest.

[82] Miyahara N, Swanson BJ, Takeda K, Taube C, Miyahara S, Kodama T, Dakhama A, Ott VL, Gelfand EW (2004). Effector CD8+ T cells mediate inflammation and airway hyper-responsiveness. Nat Med.

[83] Miyahara N, Takeda K, Kodama T, Joetham A, Taube C, Park JW, Miyahara S, Balhorn A, Dakhama A, Gelfand EW (2004). Contribution of antigen-primed CD8+ T cells to the development of airway hyperresponsiveness and inflammation is associated with IL-13. J Immunol.

[84] Tang Y, Guan SP, Chua BY, Zhou Q, Ho AW, Wong KH, Wong KL, Wong WS, Kemeny DM (2012). Antigen-specific effector CD8 T cells regulate allergic responses via IFN-gamma and dendritic cell function. J Allergy Clin Immunol.

