# Supplementary figures and images for: CD4+ and CD8+ T cells play a central role in a HDM driven model of allergic asthma
Source: Respir Res. 2016 Apr 25;17:45. doi: 10.1186/s12931-016-0359-y (PMC4845490; doi:10.1186/s12931-016-0359-y)

Additional file 1: Figure S1


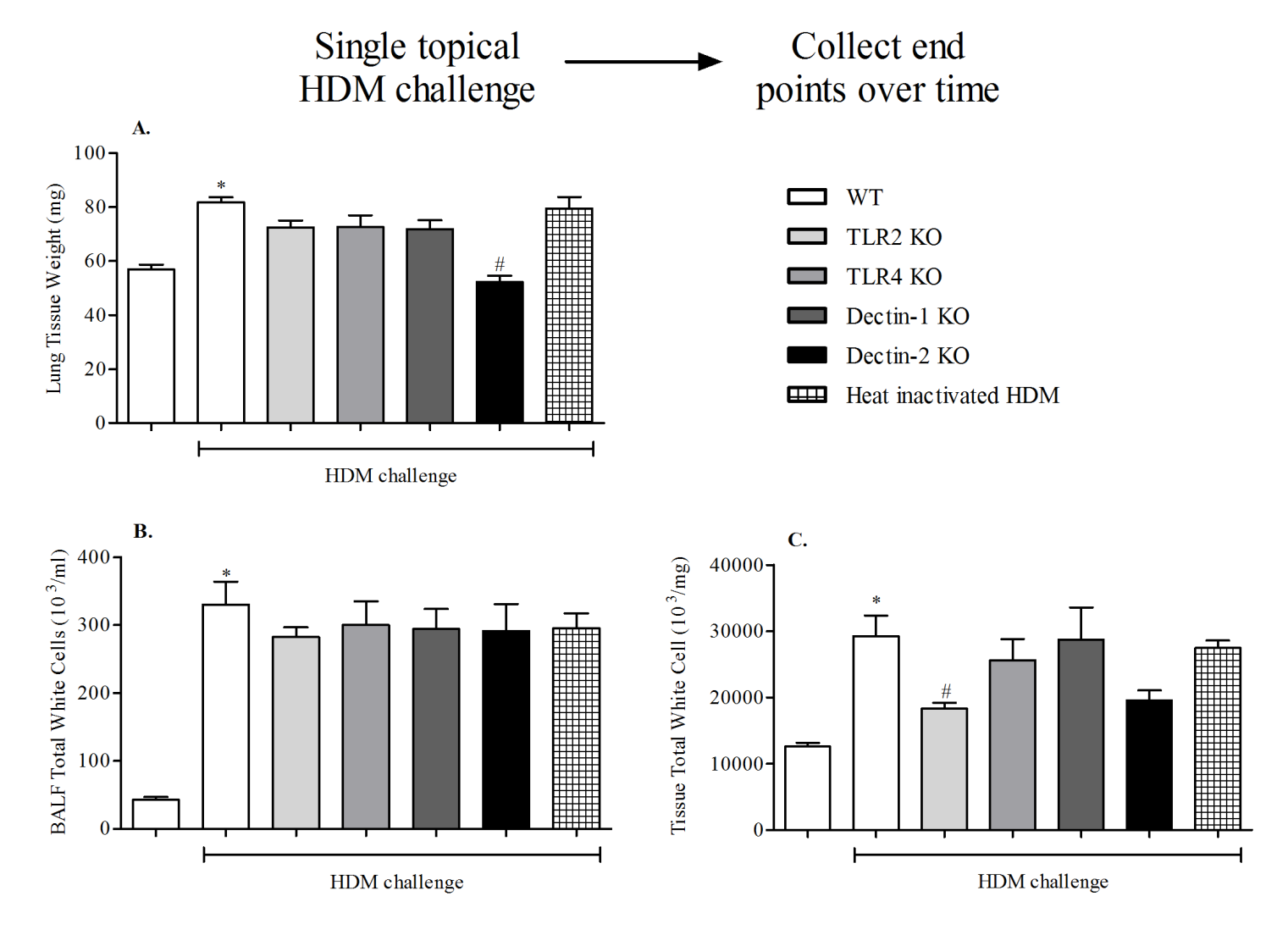

Supplement: Additional file 1: Figure S1. — Investigation of mechanism driving acute inflammatory response to HDM challenge. Naïve male C57bl/6, TLR2, TLR4, Dectin-1 and Dectin-2 KO mice were anaesthetised (4 % isoflurane in oxygen for 3 min) and challenged with i.t. vehicle (saline) or HDM (25 μg/mice in 50 μl saline). 48 h after challenge the lungs were lavaged, lung weight (A) determined, BALF (B) and lung tissue (C) total white blood cell numbers (D) were determined. Data (n = 7–8) expressed as means ± S.E.M. *p < 0.05 vs saline challenged controls (Mann-Whitney test). #p < 0.05 vs. challenged WT controls, Mann-Whitney U-test. (DOC 185 kb) [file 12931_2016_359_MOESM1_ESM.doc]

Additional file 2: Figure S2


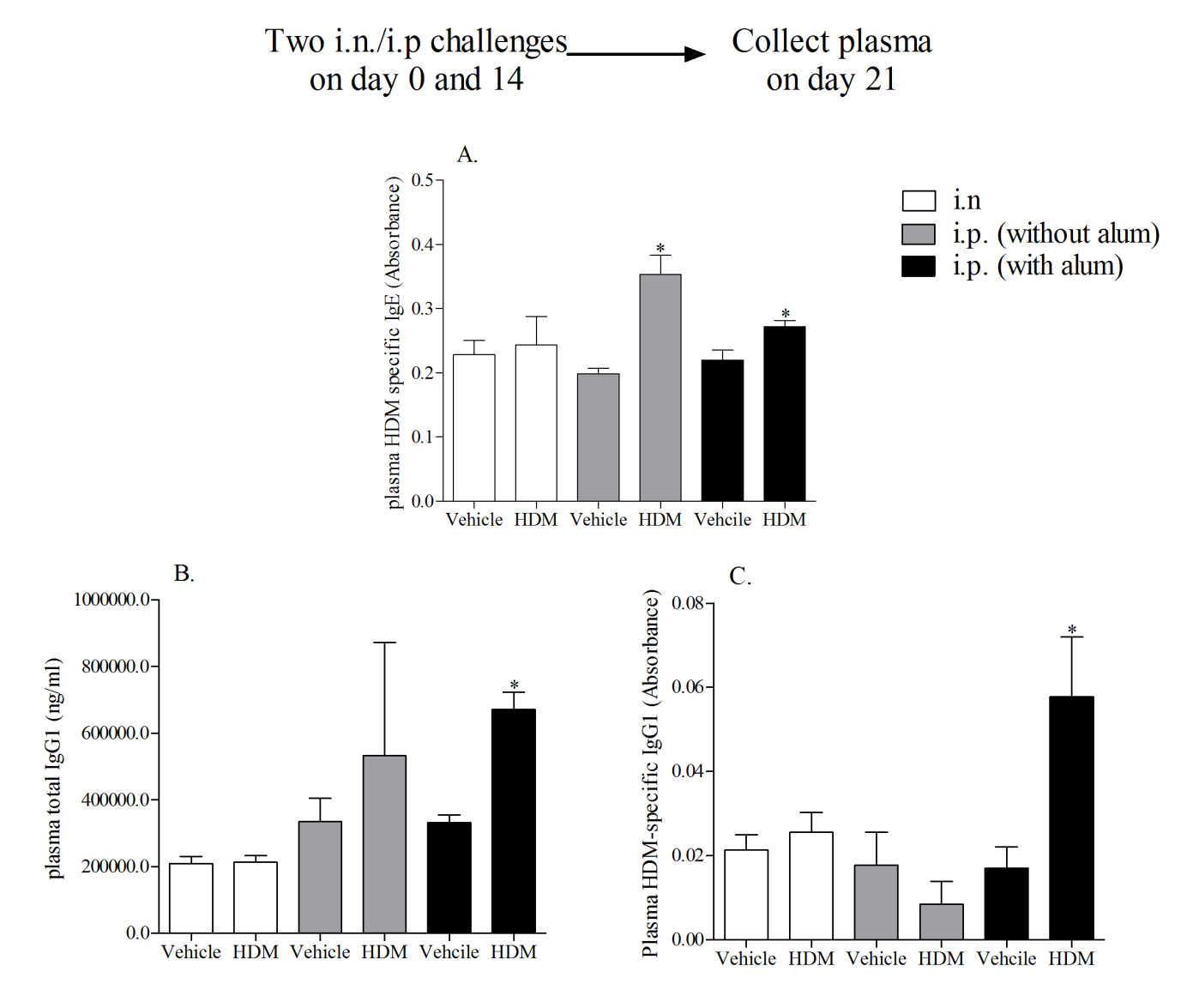

Supplement: Additional file 2: Figure S2. — Ig levels in response to sensitisation with HDM via different routes. Male C57bl/6 mice were sensitised with vehicle or HDM (5 μg/kg HDM) either i.n. or i.p. (with or without Alum). Levels of HDM-specific IgE (A), total IgG1 (B) and HDM-specific IgG1 (C) in plasma were measured by ELISA. Data (n = 6) expressed as means ± S.E.M. *p < 0.05 vs. respective saline challenged controls, Mann-Whitney U-test. (DOC 183 kb) [file 12931_2016_359_MOESM2_ESM.doc]

Additional file 3: Figure S3


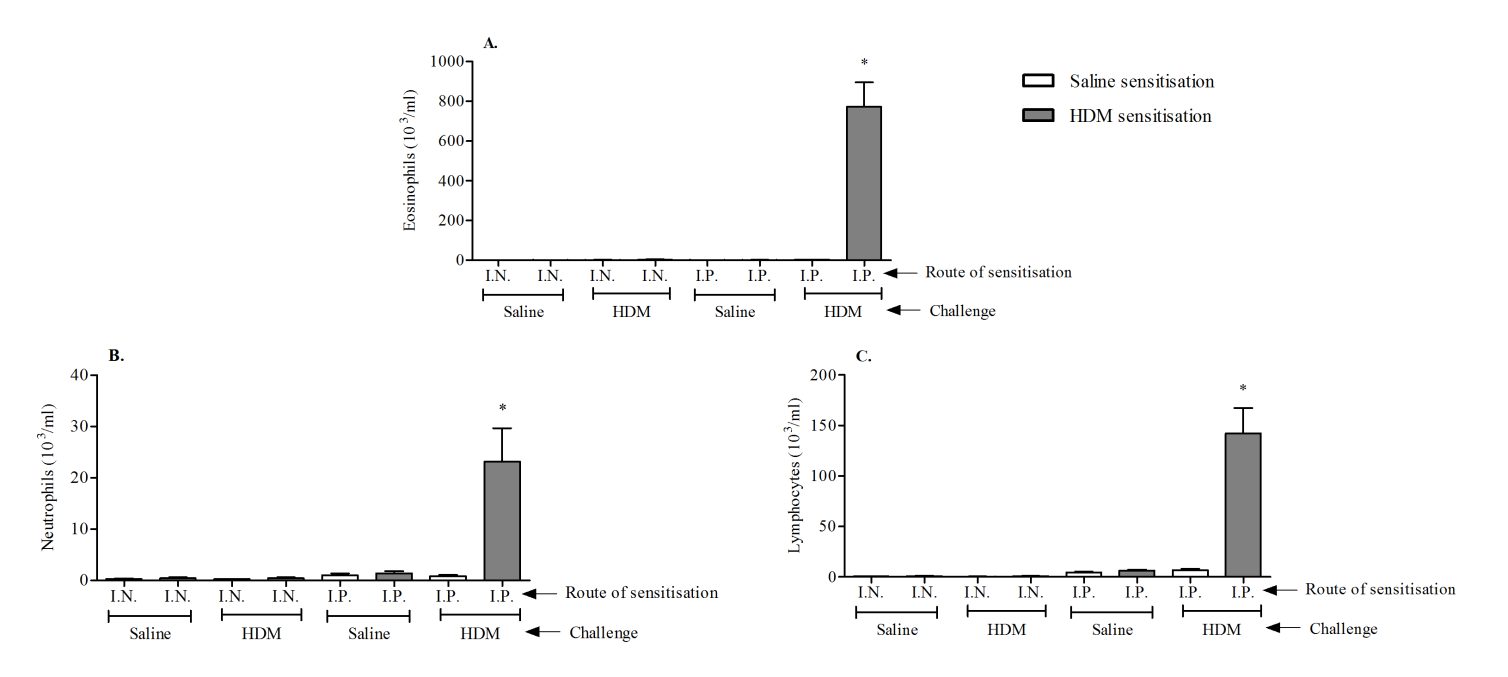

Supplement: Additional file 3: Figure S3. — The effect of intranasal HDM challenge in topically sensitised mice. Male C57bl/6 mice were sensitised i.n. or i.p. as indicated below the figures with saline or HDM. Mice were subsequently challenged with saline or HDM. 3 days after the last challenge lungs were lavaged and BALF eosinophil (A), neutrophil (B) and lymphocyte (C) numbers were determined. Data (n = 8–12) expressed as mean cell number (103/ml) ± S.E.M. *p < 0.05 vs. relevant saline sensitised controls, Mann-Whitney U-test. (DOC 109 kb) [file 12931_2016_359_MOESM3_ESM.doc]

Additional file 4: Figure S4


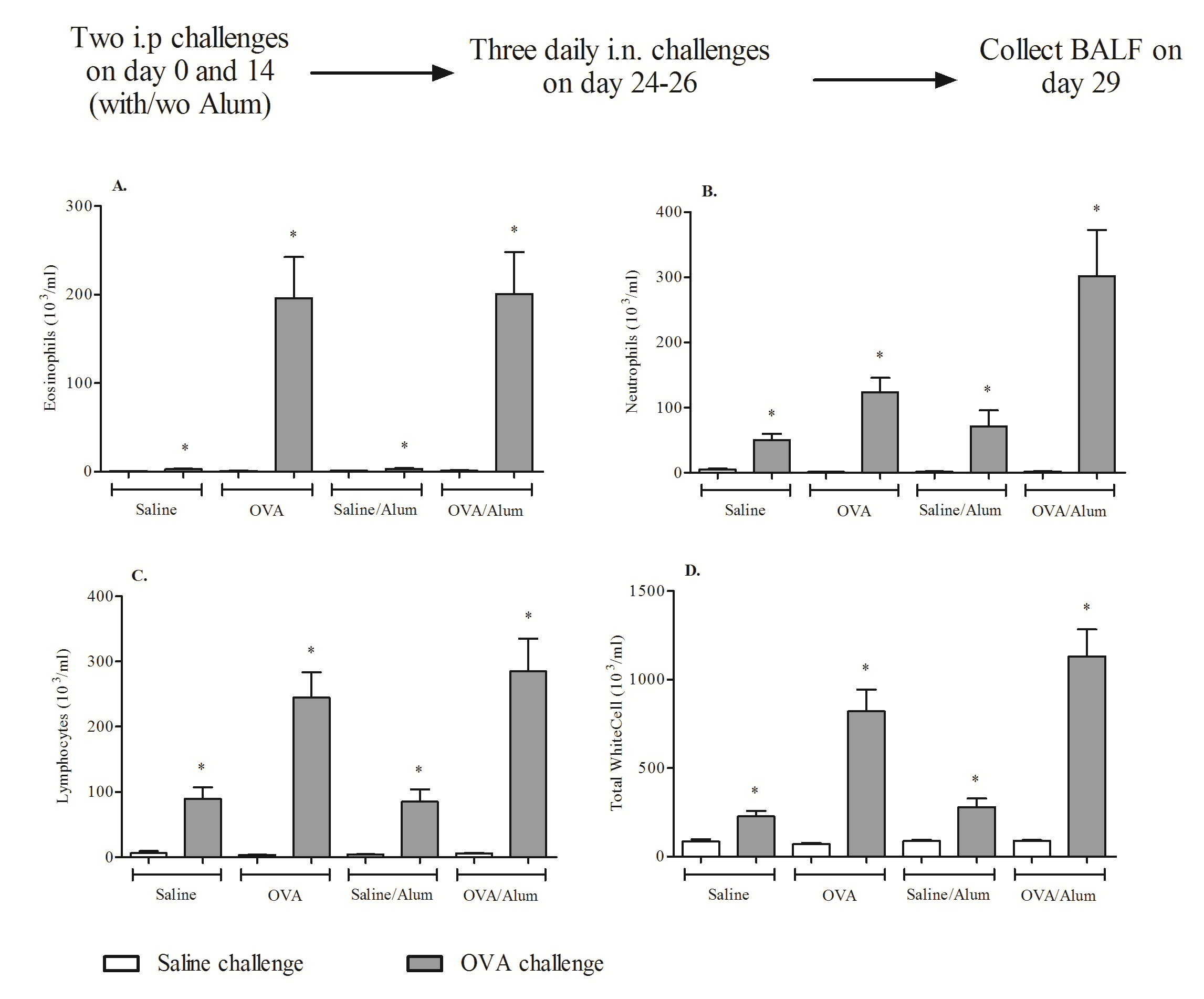

Supplement: Additional file 4: Figure S4. — The requirement for Alum during sensitisation in the allergic inflammatory response to OVA challenge. Male C57bl/6 mice were sensitised with saline or OVA made up in saline or Alum as indicated below the figure. Mice were subsequently challenged with saline (white bars) or OVA (grey bars). 3 days after the last challenge lungs were lavaged and BAL fluid eosinophil (A), neutrophil (B), lymphocyte (C) and total white blood cell (D) numbers were determined. Data expressed as mean cell number (103/ml) ± S.E.M. (n = 7–9). *p < 0.05 vs. relevant saline challenged controls, Mann-Whitney U-test. (DOC 177 kb) [file 12931_2016_359_MOESM4_ESM.doc]

Additional file 5: Figure S5


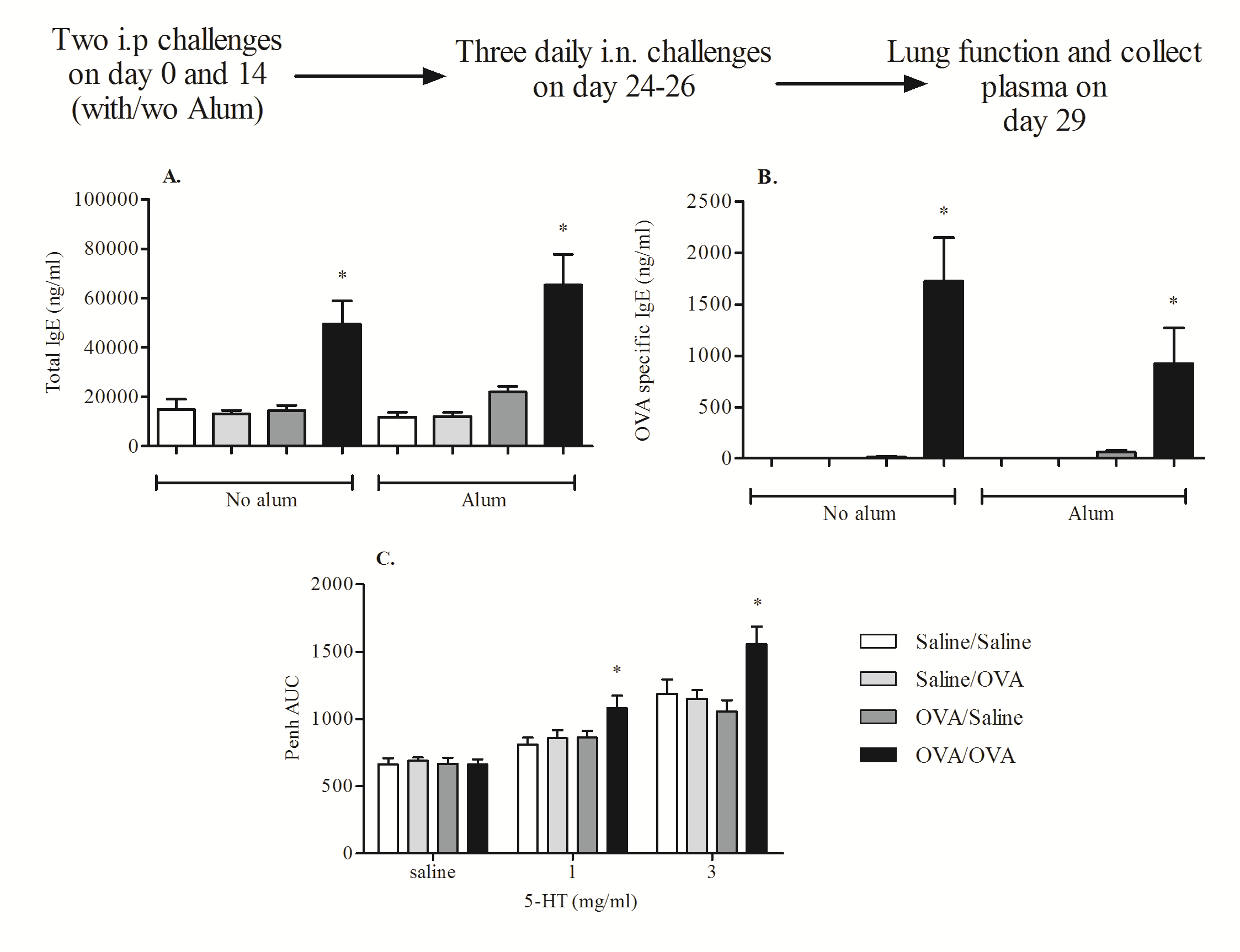

Supplement: Additional file 5: Figure S5. — Alum-independent OVA-induced airway hyperresponsiveness to 5-HT and IgE production. Male C57Bl/6 mice were sensitised with saline or OVA in the presence or absence of Alum and subsequently challenged with saline or OVA. Animals sensitised in the absence of Alum were placed in WBP chambers and airway responsiveness to 5-HT was assessed 3 days after final challenge (C). Data (n = 7–9) was expressed as mean Penh AUC ± S.E.M. Plasma levels of total IgE (A) and OVA-specific IgE (B) were assessed by ELISA 3 days after final OVA challenge. Data (n = 7–9) expressed as mean ± S.E.M. *p < 0.05 vs. relevant OVA sensitised/saline challenged controls, Mann-Whitney U-test. (DOC 168 kb) [file 12931_2016_359_MOESM5_ESM.doc]

Additional file 6: Figure S6


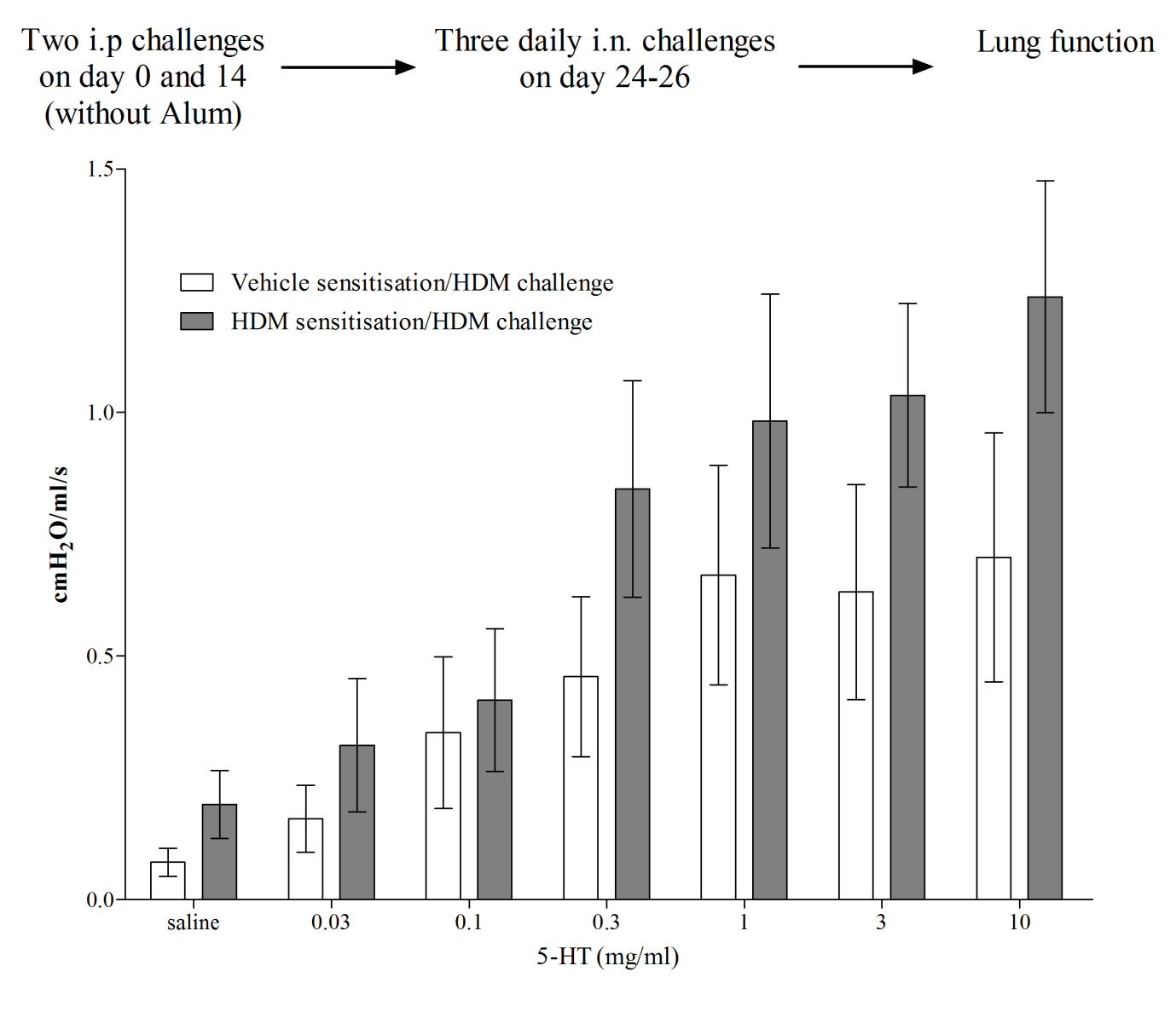

Supplement: Additional file 6: Figure S6. — Effect of HDM sensitisation and challenge on airway responsiveness to inhaled 5-HT. Male C57bl/6 mice were sensitised with saline or HDM (no Alum) and subsequently challenged with saline or HDM. Anaesthetised mice were instrumented and airway responsiveness to inhaled 5-HT was assessed by changes in resistance. Data (n = 6) expressed as mean resistance ± S.E.M. (DOC 150 kb) [file 12931_2016_359_MOESM6_ESM.doc]

Additional file 7: Figure S7


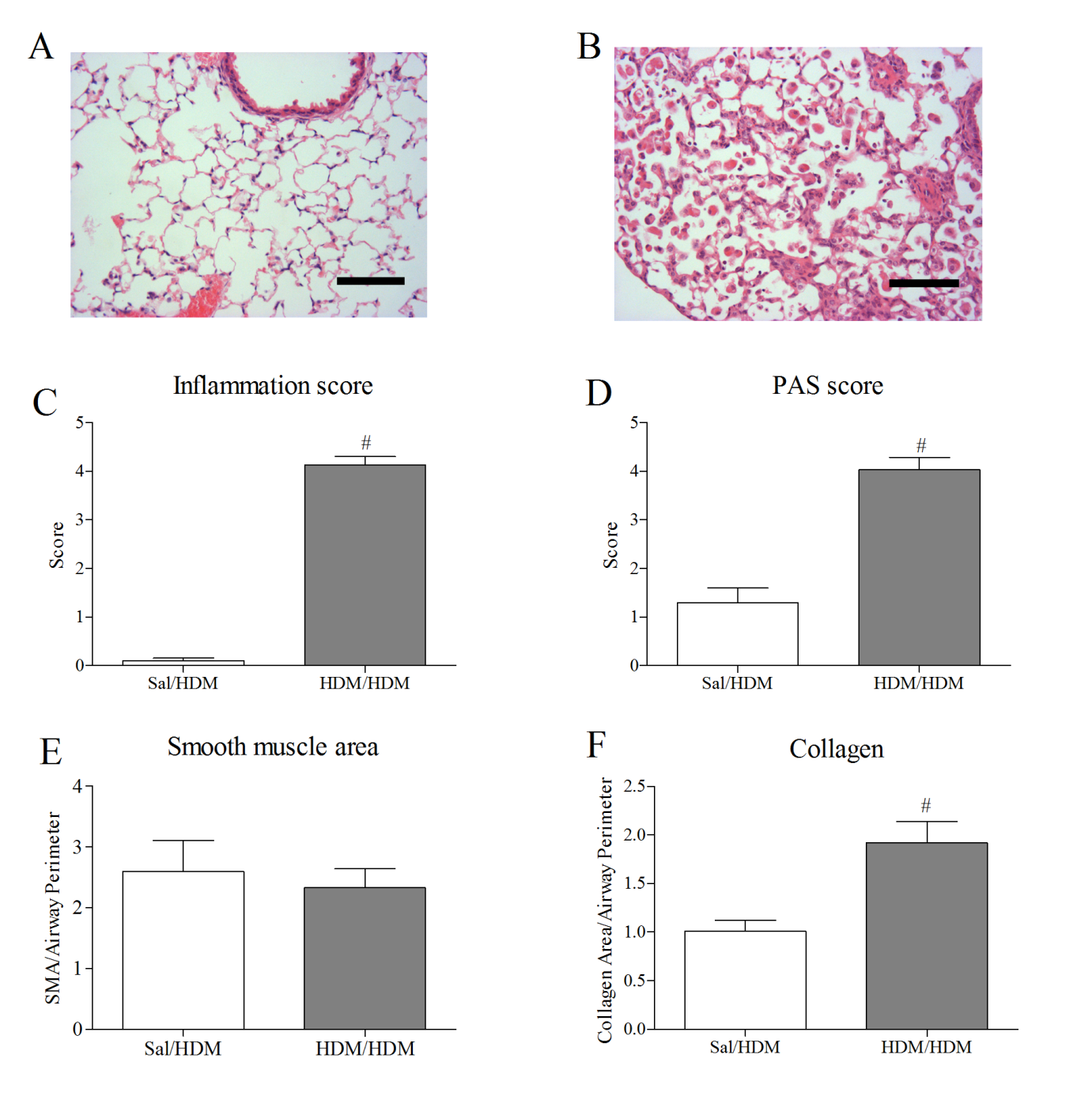

Supplement: Additional file 7: Figure S7. — The effect of chronic HDM exposure on airway pathology. Male WT C57bl/6 mice were sensitised with saline or HDM and subsequently challenged with HDM 3 times a weeks for 5 weeks. Mice were culled 3 days after final HDM challenge and the lungs collected and assessed for inflammatory status: A) example H&E staining from the control group; B) example H&E staining from the allergic HDM challenged group; C) inflammatory cell burden score; D) mucus production (PAS staining) score; E) collagen deposition (Sirius Red staining) and F) airway smooth muscle area (alpha acting staining). Data (n = 6) expressed as mean ± S.E.M. #p < 0.05, Mann-Whitney U-test. (DOC 940 kb) [file 12931_2016_359_MOESM7_ESM.doc]
